# Simultaneously targeting SOAT1 and CPT1A ameliorates hepatocellular carcinoma by disrupting lipid homeostasis

**DOI:** 10.1038/s41420-021-00504-1

**Published:** 2021-05-29

**Authors:** Meiling Ren, Huanji Xu, Hongwei Xia, Qiulin Tang, Feng Bi

**Affiliations:** Department of Abdominal Oncology, Cancer Center and Laboratory of Molecular Targeted Therapy in Oncology, West China Hospital, Sichuan University, Chengdu, Sichuan Province 610041 China

**Keywords:** Targeted therapies, Cancer metabolism, Lipid signalling

## Abstract

Lipid homeostasis plays a fundamental role in the development of hepatocellular carcinoma (HCC). However, the mechanisms that regulate lipid homeostasis to avoid lipotoxicity in HCC remain elusive. Here, we found high-fat diet (HFD) improved the expression of sterol o-acyltransferase1 (SOAT1) and carnitine palmitoyltransferase 1A (CPT1A) in diethylnitrosamine-induced HCC. Bioinformatic analysis showed that SOAT1-mediated fatty acid storage and CPT1A-mediated fatty acids oxidation (FAO) formed a double-negative feedback loop in HCC. We verified that SOAT1 inhibition enhanced CPT1A protein, which shuttled the released fatty acids into the mitochondria for oxidation in vivo and in vitro. Besides, we further confirmed that CPT1A inhibition converted excess fatty acids into lipid drops by SOAT1 in vitro. Simultaneously targeting SOAT1 and CPT1A by the small-molecule inhibitors avasimibe and etomoxir had synergistic anticancer efficacy in HCC in vitro and in vivo. Our study provides new mechanistic insights into the regulation of lipid homeostasis and suggests the combination of avasimibe and etomoxir is a novel strategy for HCC treatment.

## Introduction

Cancer cells undergo characteristic metabolic changes to adapt to their local environment, so-called “metabolic reprogramming^1^”. Among the most well-known studies of these changes is the Warburg effect^2^, in which cancer cells highly rely on aerobic glycolysis to obtain energy rather than mitochondrial oxidative phosphorylation even in the presence of oxygen. Altered fatty acids metabolism is another property of cancer cells^3^. Tumor cells exhibit abnormal fatty acid biosynthesis^4^ and uptake to meet their high energy requirements associated with rapid tumor growth. However, a sustained overload of free fatty acids (FFAs) disturbs cellular functions^5^ or even causes cytotoxicity, leading to cell death, which is known as lipotoxicity^6–9^. Controlling FFAs levels and maintaining their homeostasis is crucial in cancer development, especially in steatohepatitis hepatocellular carcinoma (SH-HCC), a subcategory of HCC characterized by HCC with steatosis. In the obese, increased FFA intake along with visceral adipose tissue lipolysis leads to the accumulation of enormous exogenous FFAs in the HCC microenvironment. This lipid-enriched condition is a very characteristic environment for liver cancer. But how the malignant cells maintain lipid homeostasis for their progression and prevent lipotoxicity is less explored.

Lipid homeostasis is regulated by a complex mechanism, including their input (biosynthesis and uptake) and export (storage as lipid droplets, oxidization, conversion, and excretion). When cellular FFAs are in excess, SOAT1 and SOAT2 catalyzed FFAs to form cholesterol esters (CEs), which are then assembled and bud from the endoplasmic reticulum (ER) into the cytosol to form lipid drops (LDs)^10^. Besides, non-esterified FFAs are used for energy generation in fatty acid β-oxidation (FAO), which sustains tumor growth^11,12^. CPT1, a rate-limiting enzyme in FAO, transferred FFAs into the mitochondrial matrix and oxidized them to acetyl-CoA^13^. FAO-derived acetyl-CoA can be further oxidized to ATP via trichloroacetic acid (TCA) cycle or converted to ketone bodies through ketogenesis. Ketone bodies generated from FAO act as an alternative source of energy when glucose supply is depleted and promote tumor progression^14^. Previous studies have shown that SOAT1 or CPT1 was over-expressed in many cancers^15^, including breast cancer, HCC, and GBM, making it a potential metabolic target in cancer treatment^16,17^. Fatty acid esterification and oxidation play an important role in tumor growth and blocking one of the fatty acid degradation pathways alone can suppress tumor growth^8,18,19^. However, there is a paucity of research evaluating the two fatty acid degradation pathways as a whole.

This study revealed the interaction between SOAT1-mediated fatty acid storage and CPT1A-mediated FAO is essential for lipid homeostasis and provides a new approach for lipid metabolic-targeted HCC treatment.

## Results

### A high-fat diet (HFD) improves the expression of SOAT1 and CPT1A in DEN-induced HCC

**Fig. 1:**
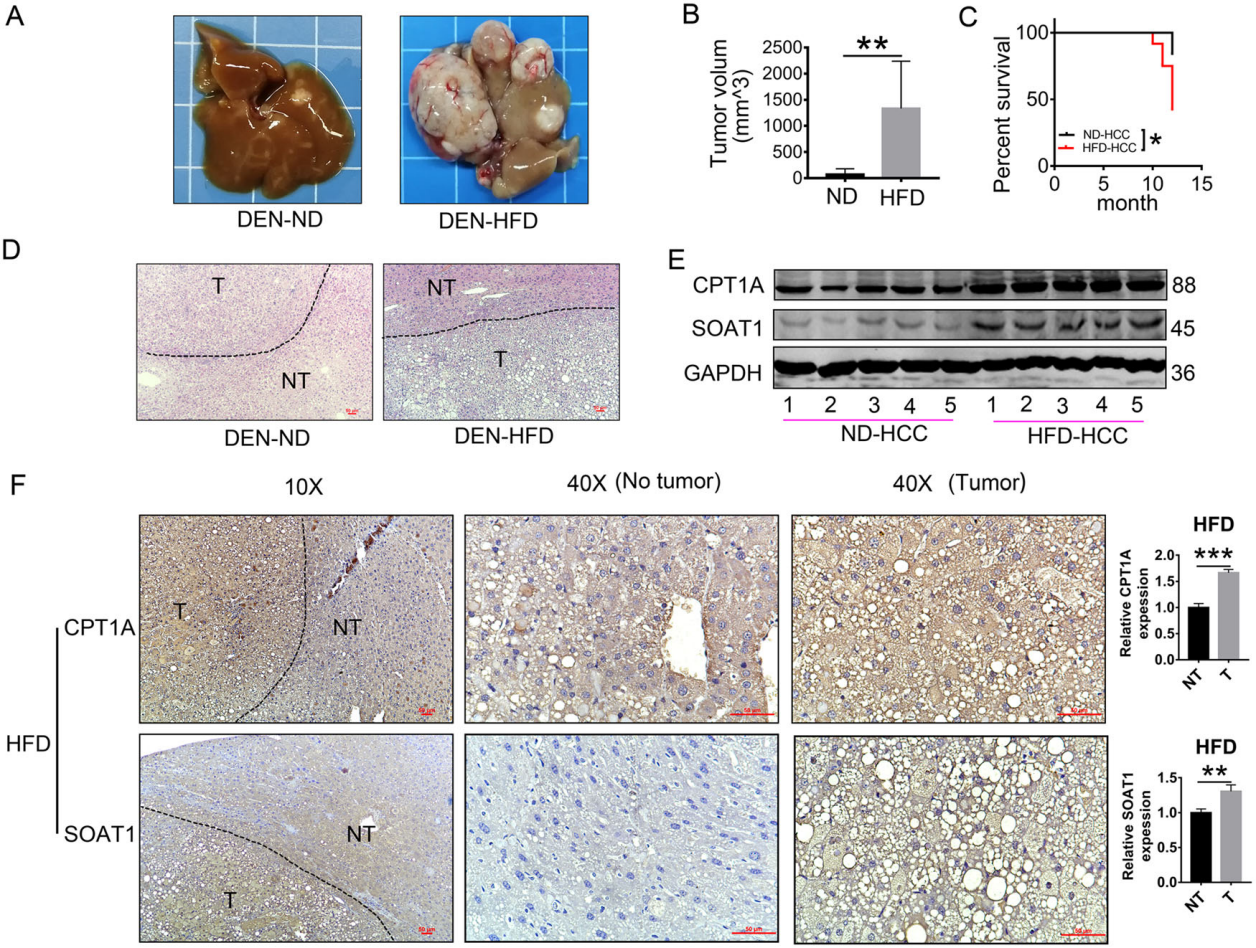
HFD improves the expression of SOAT1 and CPT1A in DEN*-*induced HCC. **A**–**B** A high-fat diet exacerbated HCC progression in DEN-injected mice. Representative images of the livers (**A**) and tumor volumes (**B**), ND: the normal diet group (*n* = 8) and HFD: the high-fat diet group (*n* = 5). (mean ± SEM) **p < 0.01. **C** Kaplan–Meier survival plot of DEN-injected mice on a high-fat diet (HFD) or normal diet (ND); *p < 0.05. **D** H&E-stained images of livers using HCC and adjacent non-tumor liver tissue in ND-fed or HFD-fed mice (scale bar, 50 μm). NT and T indicated non-tumor and tumor areas, respectively. **E** Western blot analysis of SOAT1 and CPT1A in liver cancer tissues from ND-fed or HFD-fed mice. **F** Representative IHC staining (*n* = 5 images in total) of SOAT1 or CPT1A in the tissues obtained from HFD-fed mice. NT and T indicate non-tumor and tumor areas, respectively (scale bar, 50 μm). Five separate areas from each tissue were quantified (mean ± SEM); *p < 0.05; **p < 0.01, ***p < 0.001.

Consistent with the previous studies^20^, we found that HFD accelerated the progression of DEN-induced HCC. Images of the livers and tumor volumes from each group of mice were shown in Fig. 1A and B. Kaplan–Meier curves illustrated HFD shortened the mice’s survival (Fig. 1C). HFD-fed mice exhibited more prominent tumor steatosis in HCC than in adjacent non-tumor tissues and HCC tissues of ND-fed mice in H&E staining (Fig. 1D). Marked steatosis was a common feature among the HFD-promoted HCC tissues, but how the malignant cells maintain lipid homeostasis for their progression and prevent lipotoxicity is less explored. We analyzed the expression of SOAT1 and CPT1A, the genes related to fatty acid degradation. Western blot analysis revealed HFD elevated the expression of SOAT1 and CPT1A in HCC (Fig. 1E). Immunohistochemistry (IHC) analysis was used in paired non-tumor (NT) and HCC tissues obtained from DEN-injected mice on a normal diet (ND) or a HFD (ND-NT, ND-HCC, HFD-NT, and HFD-HCC tissues). The result showed the expressions of SOAT1 and CPT1A were mildly decreased under a ND condition (Fig. S1), but dramatically increased under HFD condition in HCC tissues compared with the adjacent non-tumor tissues (Fig. 1F). These findings indicated that the overexpression of SOAT1 and CPT1A was independent of DEN administration and played an important role in HFD-promoted HCC.

How did HFD enhance the expression of SOAT1 and CPT1A? We assumed fatty acid can increase the expression of SOAT1 and CPT1A. To verify the idea, we found a low concentration of palmitic acid can enhance the expression of SOAT1 (Fig. S2). Moreover, palmitic acid and oleic acid increased the expression of CPT1A and HMGCL, the enzymes of FAO and ketogenesis (Fig. S3A–B). Palmitic acid also enhanced the production of acetyl-CoA, ATP, and ketone bodies, the metabolites of FAO, TCA cycle, and ketogenesis (Fig. S3, C–F) in vitro; A similar observation was also reported in a previous study in which fatty acids activate mitochondrial fatty acid oxidation to promote tumor growth in colon cancer^21^, whereby fatty acids activate the pathways of SOAT1-mediated fatty acid storage and CPT1A-mediated FAO. In the subsequent experiments, we investigated the mechanism and function of SOAT1 and CPT1A in HCC.

### SOAT1, but not SOAT2, has more potential role in the development of HCC and negatively correlated with the FAO pathway on data mining

**Fig. 2:**
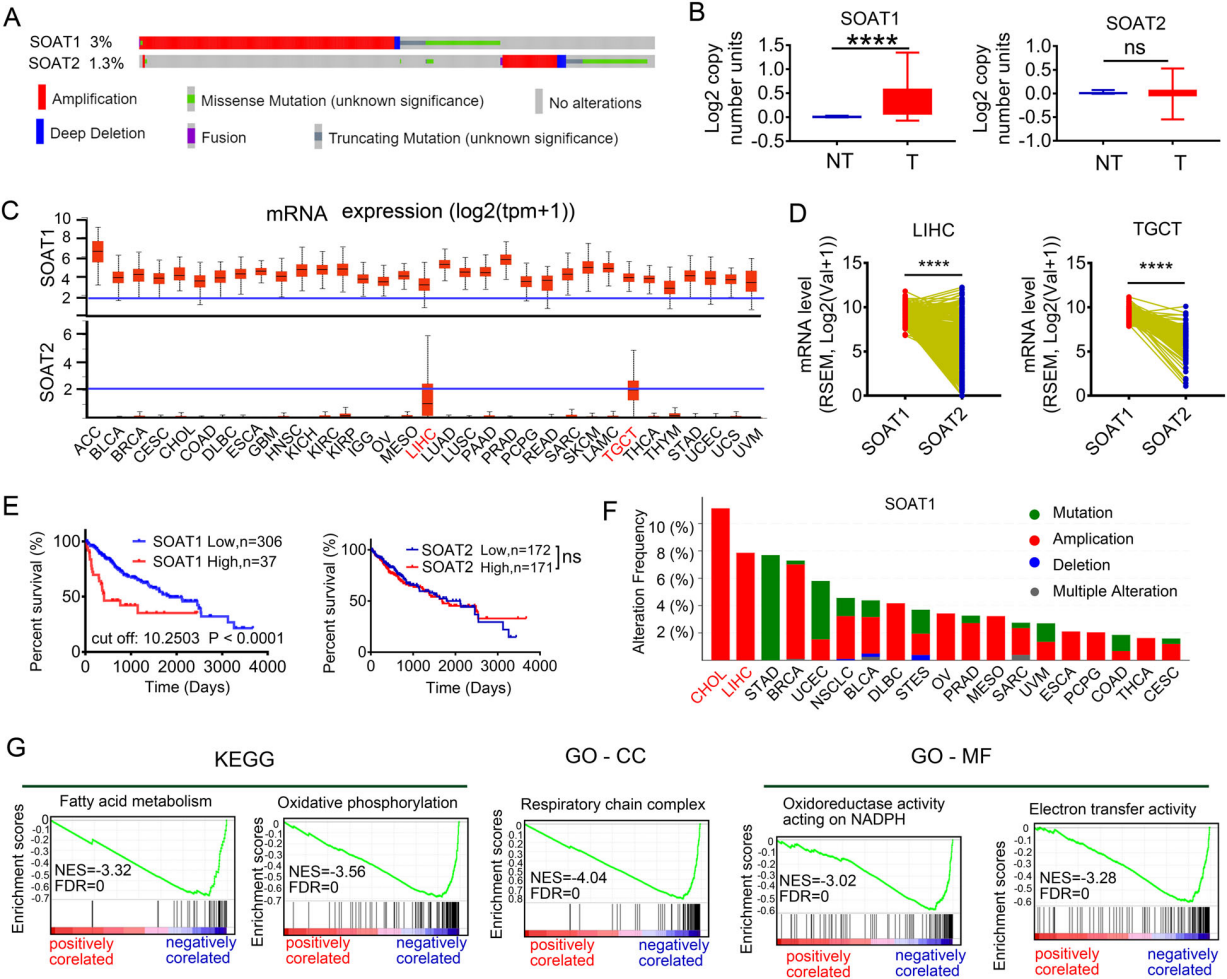
SOAT1, but not SOAT2, has a more important role in the development of HCC and is negatively correlated with FAO pathway on data mining. **A** Frequency and types of SOAT1 and SOAT2 DNA alteration in TCGA using cBioPortal. **B** Copy number variation of SOAT1 or SOAT2 in HCC and normal tissue. ****p < 0.0001; ns, no significance. **C** mRNA expression pattern of SOAT1 (up) vs. SOAT2 (bottom) in 30 cancer types in the TCGA database. **D** Paired profiles of SOAT1 vs SOAT2 mRNA expression in tumor tissues from individuals with HCC and TGCT in the TCGA database. RSEM, RNA-Seq by Expectation Maximization. **E** Kaplan–Meier survival plot of HCC patient based on SOAT1 or SOAT2 expression using TCGA database. SOAT1 cutoff is 10.2503. SOAT2 cutoff is 8.109. Significance was analyzed by Log-rank test. **F** The frequency of SOAT1 DNA alteration in 19 cancer types from the TCGA database. **G** Enrichment analysis of the genes altered in the SOAT1 neighborhood in HCC, BP biological processes, MF molecular functions, CC cellular component, KEGG KEGG pathway analysis, NES normal enrichment score.

SOATs family include SOAT1 and SOAT2. We firstly evaluated the DNA alterations of SOAT1 and SOAT2 in the Cancer Genome Atlas (TCGA) pan-cancers via cBioPortal. The frequencies were 3% and 1.3% for SOAT1 and SOAT2 alterations, respectively. Besides, amplification was the most common type of SOAT1 alterations (Fig. 2A). And the copy number variation of SOAT1 was elevated in tumor tissues compared with non-tumor tissues, while SOAT2 had no significance using the Oncomine database (Fig. 2B). Consistent with the DNA alteration, SOAT1 mRNA level (up) was dramatically higher than SOAT2 (bottom) from UNICAL (Fig. 2C). We further examined mRNA expressions of SOAT1 vs. SOAT2 in liver hepatocellular carcinoma (LIHC) and testicular germ cell tumors (TGCT) tissues; the result revealed that SOAT1 expression remained higher than SOAT2 (Fig. 2D). Moreover, the high expression of SOAT1 was associated with poor prognosis in patients with HCC, while SOAT2 had no significance (Fig. 2E). The two cancer types bearing the highest SOAT1 alteration frequency in TCGA pan-cancers were cholangiocarcinoma (CHOL) and LIHC, both of which belong to liver cancer (Fig. 2F). Those implied that SOAT1 can serve as a more potential diagnostic indicator in HCC compared with SOAT2. Then we used LinkedOmics to investigate the biological function of SOAT1 in HCC from 371 LIHC patients in the TCGA. Gene set enrichment analysis (GSEA) showed that the genes negatively correlated with SOAT1 were mainly located in the fatty acid metabolism, oxidation-reduction process, and respiratory chain complex (Fig. 2G). Finally, we visualized the protein–protein interaction network of SOAT1, fatty acid metabolism, and TCA cycle using STRING database (Fig. S4).

### CPT1A, but not CPT1B and CPT1C, plays a dominant role in human HCC and correlated inversely with SOAT1 on data mining

**Fig. 3:**
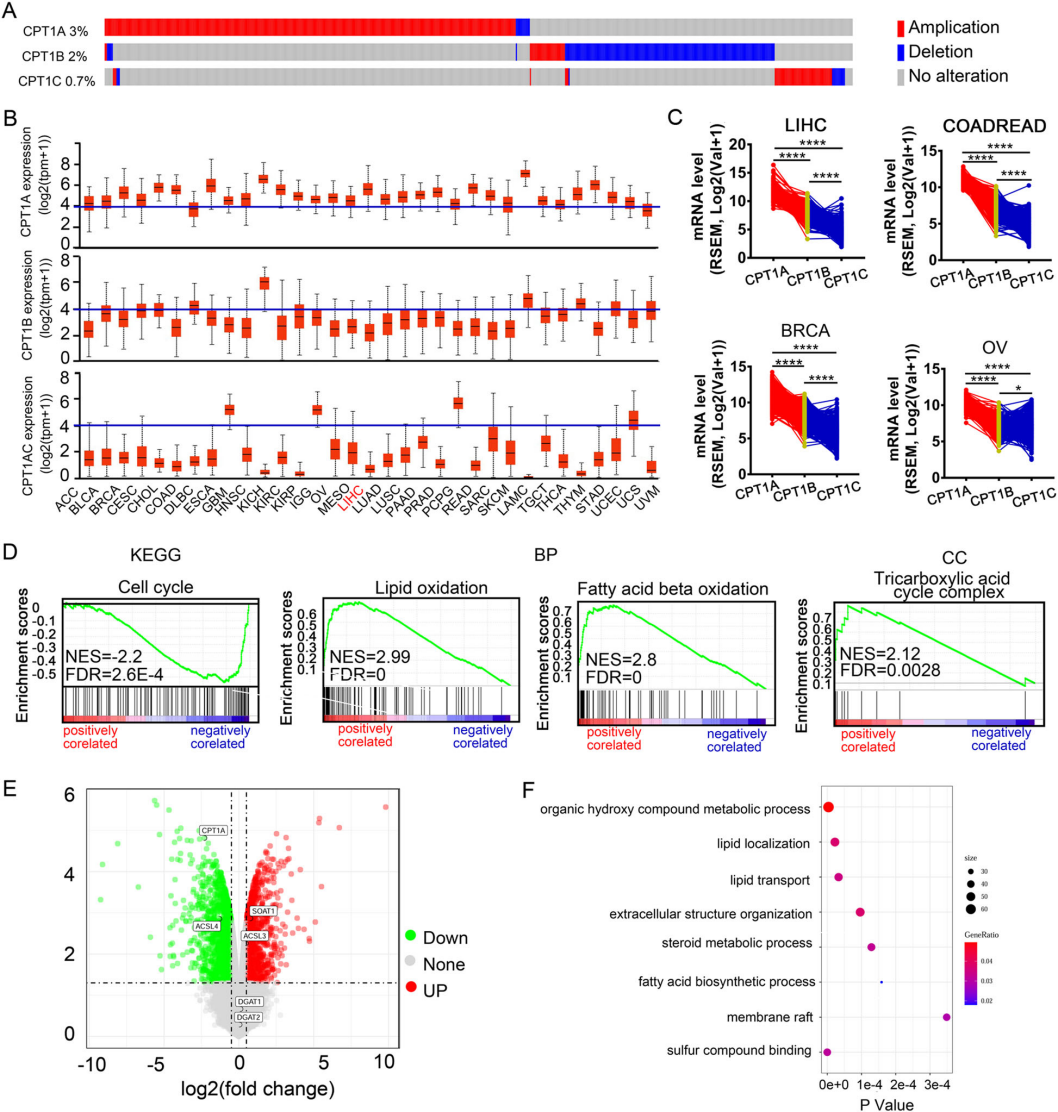
CPT1A, but not CPT1B and CPT1C, plays a dominant role in human HCC and correlated inversely with SOAT1 on data mining. **A** The DNA alterations of CPT1A, CPT1B, and CPT1C in 30 cancers using TCGA database from cBioPotal. **B** CPT1A (top), CPT1B (middle), and CPT1C (bottom) mRNA expression pattern in 30 cancer types in the TCGA database from UNICAL. **C** Paired profiles of CPT1A, CPT1B, and CPT1C mRNA expression in tumor tissues from individuals with HCC and other cancers in the TCGA database from linkedOmics. COADREAD colon adenocarcinoma and rectum adenocarcinoma, RSEM RNA-Seq by expectation maximization. **D** Enrichment analysis of the genes altered in the CPT1A neighborhood in HCC (LinkedOmics). BP biological processes, MF molecular functions, CC cellular component, KEGG KEGG pathway analysis. **E** CPT1A is inversely correlated with SOAT1. Volcano plot of differentially expressed. The red points represented upregulated genes. The green points represented down-regulated genes. **F** Gene ontology enrichment analysis of differentially expressed proteins in silencing CPT1A cell.

CPT1 family comprises three subtypes, CPT1A (liver type), CPT1B (muscle type), and CPT1C (brain type)^22^. The cBioPortal was used to determine the DNA alterations of CPT1A, CPT1B, and CPT1C in the TCGA database. The frequencies were 3%, 2%, and 0.7% for CPT1A, CPT1B, and CPT1C alterations. Amplification was the most common type of CPT1A CNV (Fig. 3A). TCGA pan-cancer analysis showed that the level of CPT1A mRNA was highest among the CPT1 family in most cancer types, except GBM (Fig. 3B–C). We concluded that CPT1A was a more potential diagnostic marker of HCC than CPT1B and CPT1C. Next, to investigate the function of CPT1A in HCC, we used LinkedOmics to analyze mRNA sequencing data from 371 LIHC patients. GO and KEGG enrichment analysis by GSEA showed that the genes positively correlated with CPT1A were located mainly in lipid oxidation and tricarboxylic acid cycle complex (TCA cycle) (Fig. 3D).

We next investigated metabolic alteration after silencing CPT1A in the GEO database (GSE83547). The result uncovered CPT1A knockdown upregulated the expression of SOAT1, but not DGAT1 or DGAT2 (the other enzymes of esterification) (Fig. 3E). Gene ontology enrichment analysis showed differentially expressed genes in silencing CPT1A cells were located mainly in lipid localization, fatty acid biosynthetic process, and membrane raft (Fig. 3F). Altogether, we speculate SOAT1 and CPT1A form a double-negative feedback loop to maintain lipid homeostasis and test this idea in the following experiments.

### SOAT1 inhibition upregulates CPT1A expression, which shuttles released FFAs into mitochondria for oxidation in vitro and in vivo

**Fig. 4:**
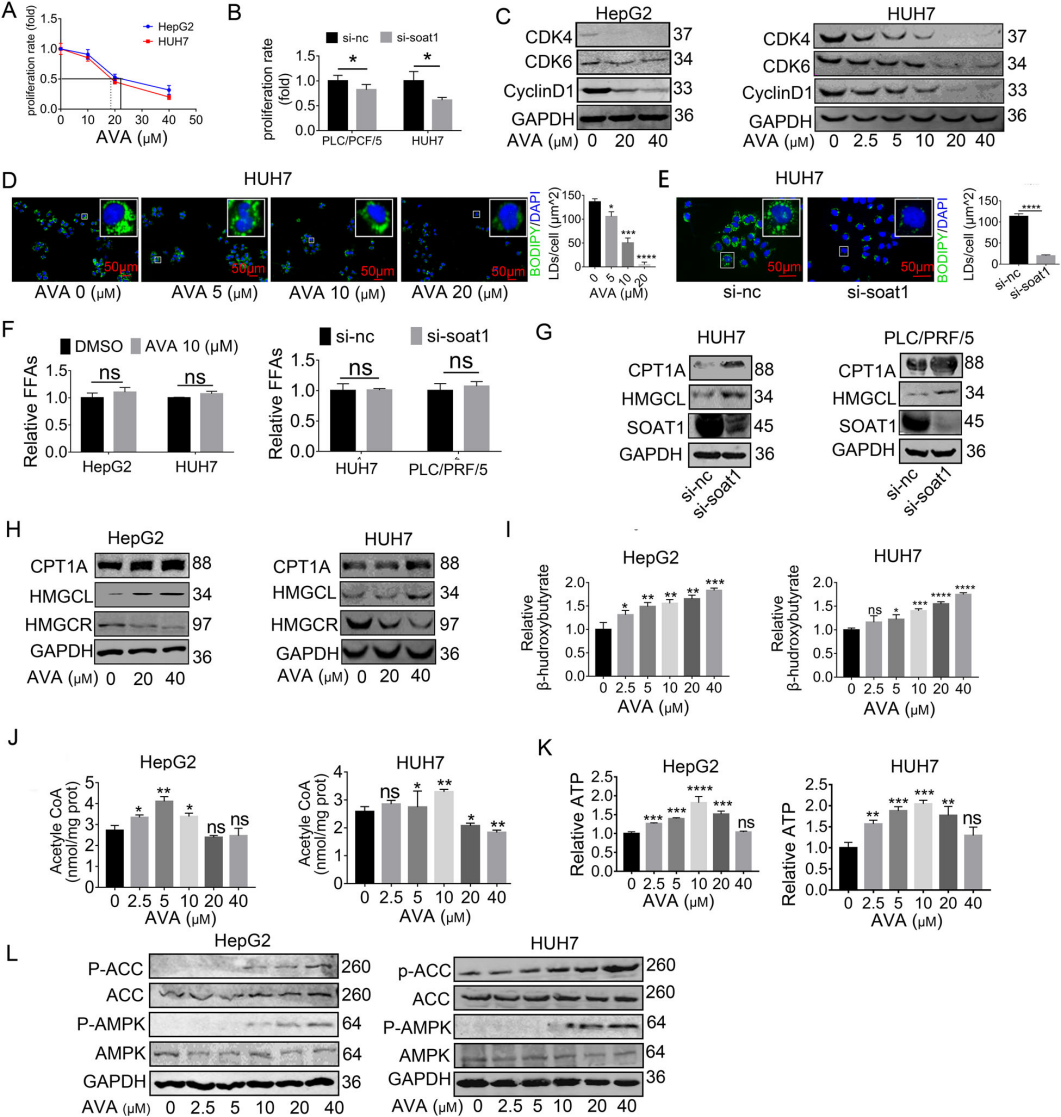
SOAT1 inhibition upregulates CPT1A expression, which shuttles released FFAs into mitochondria for oxidation in vitro. **A**–**B** CCK8 assay showed that SOAT1 knockdown inhibited cell viability in HepG2 and HUH7, (mean ± SEM, *n* = 3), *p < 0.05. IC50 values of avasimibe (AVA) were about 20 μm. **C** Western blot analysis of CDK4, CDK6, and cyclinD1 protein with AVA treatment. **D**–**E** Fluorescence imaging of LDs stained with BODIPY 493/503 (green) in HUH7 treated with pharmacological (avasimibe, 48 h) or genetic (si-RNA, 72 h) inhibition of SOAT1. Nuclei were stained with DAPI (blue). Scale bar, 50 μm. More than 30 cells were analyzed. Data were quantified using ImageJ software; *p < 0.05; ***p < 0.001, ****p < 0.0001. Significance was determined by unpaired student’s *t*-test. **F** The level of total free fatty acids in HCC cells treated with SOAT1 inhibition. (mean ± SEM, *n* = 3), ns, no significance. **G**–**H** Western blot analysis of CPT1A and HMGCL in HepG2 and HUH7 treated with pharmacologic o*r* genetic SOAT1 inhibition. **I**–**K** Levels of total acetyl-CoA, ATP, and β-hydroxybutyrate in HCC cells treated with avasimibe (AVA) for 48 h at an indicated concentration (mean ± SEM, *n* = 3); *p < 0.05; ***p < 0.001, ****p < 0.0001; ns, no significance. **L** Western blot analysis of P-ACC (S79), ACC, P-AMPK (T172), and AMPK in HCC cells treated with AVA for 48 h at the indicated concentration.

Consistent with previous studies^23–25^, targeting SOAT1 suppresses tumor growth in HCC using CCK8 assay and western blot analysis (Fig. 4A–C), but the metabolic alteration response to SOAT1-targeted therapy, which might contribute to exploring new approaches that enhance the cytotoxicity of avasimibe in HCC, is little known. Since cholesterol esterification is a major component of LDs and SOAT1 is an essential enzyme for cholesterol esterification synthesis, we sought to determine if SOAT1 is correlated with LDs formation in HCC. We first measured the levels of LDs, free cholesterol, and FFAs after SOAT1 inhibition. Consistent with the previous findings^23,26,27^, our result indicated SOAT1 inhibition rapidly blocked LDs formation (Fig. 4D–E) and enhanced the level of cellular free cholesterol (Fig. S5). Unexpectedly, we did not observe any significant accumulation in FFAs in SOAT1 knockdown cells (Fig. 4F), contrary to our original thought that SOAT1 inhibition would elevate FFAs. Previous studies showed fatty acids promoted FAO^8^; we therefore hypothesized that the released FFAs were shuttled into mitochondria for oxidation and energy production. Based on our data mining, although single SOAT1 inhibition indeed reduced cell proliferation in HCC, the anti-cancer effect could be counteracted by provoking the activity of the FAO pathway. To test the idea, we measured the FAO-related enzymes and metabolites in HCC cells exposed to avasimibe. We found SOAT1 inhibition significantly upregulates the CPT1A protein, which can facilitate the entry of excessive FAs into the mitochondria for oxidation, leading to a remarkable rise in acetyl-CoA production. As FAO-derived acetyl-CoA can be further synthesized into ketone bodies through HMG-CoA lyase (HMGCL), the enzyme of ketogenesis, we then measured protein HMGCL and ketone bodies. The result uncovered avasimibe caused continuous elevation of HMGCL and ketone bodies (Fig. 4G–J, Fig. S6). However, ATP generated from the TCA cycle first increased and then decreased with increasing avasimibe dosages. The maximum appeared at the concentration of 10 μM (Fig. 4K). Besides, the AMPK-ACC pathway was activated when avasimibe’s concentration exceeded 10 μM, in which concentration ATP began to reduce (Fig. 4L). Reports have implicated the reduced ATP stimulated AMPK*/*ACC/CPT1A axis^28^. That partly explained how avasimibe amplifies the FAO pathway and ketogenesis via a positive-feedback loop in vitro. Since the FAO alteration after avasimibe (AVA) treatment was similar to that after PA treatment and fatty acids promoted FAO^18^, we further examined whether inhibiting SOAT1 upregulate CPT1A by FFAs. The intracellular LDs were time*-*dependently decreased after serum starvation and reached the lowest values at day 3 (Fig. S7A). Of note, targeting SOAT1 no longer reduced the LDs content in the 3-day*-*starved cells (Fig. S7B), which also indicated no more fatty acid could be released into the hepatic cytoplasm. Moreover, Western blot showed avasimibe did not upregulate CPT1A expression in HCC cells after serum starvation for 3 days (Fig. S7C). Overall, targeting SOAT1 upregulated CPT1A through FFAs.

**Fig. 5:**
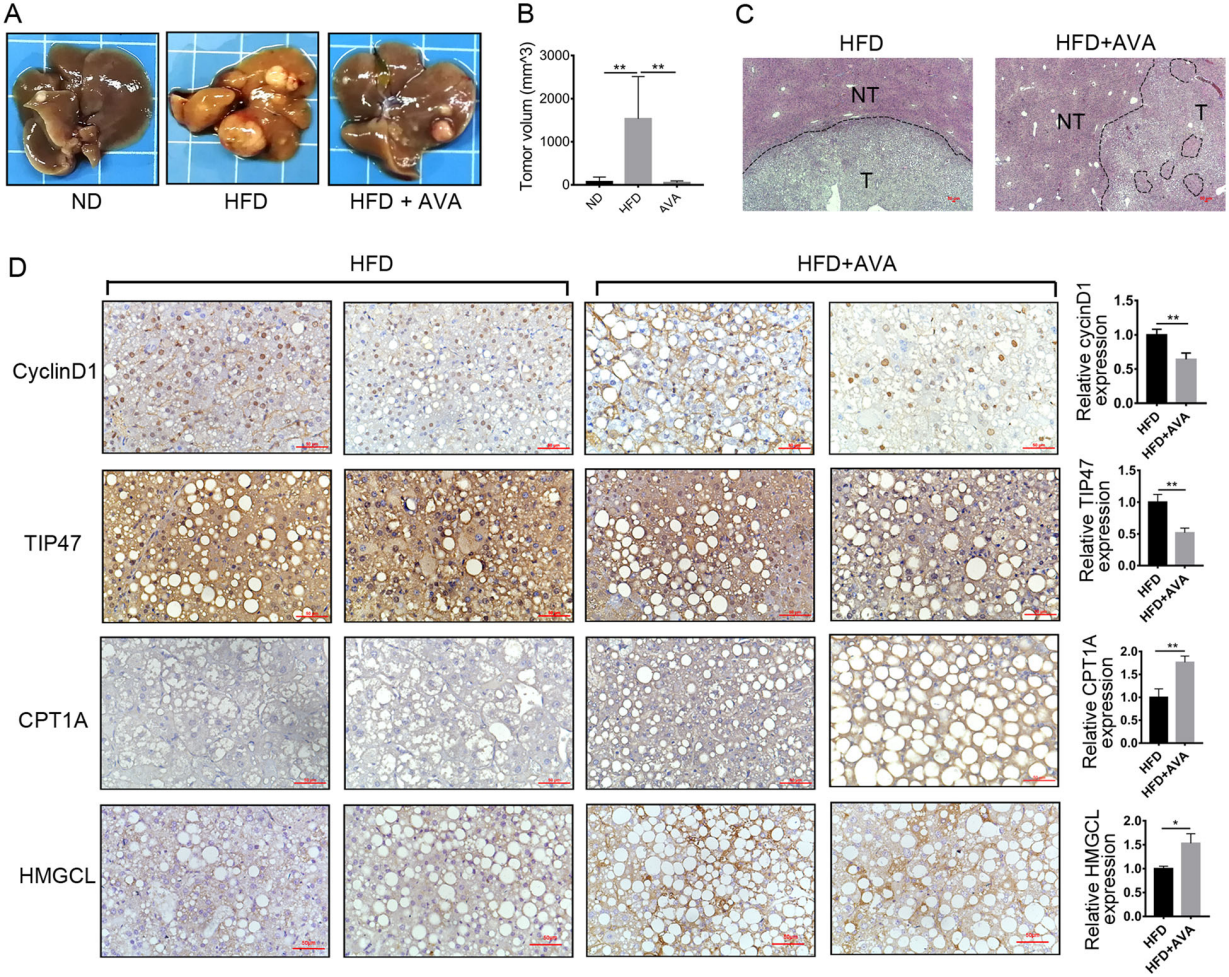
Avasimibe suppressed tumor growth but enhances CPT1A expression in DEN-injected mice on HFD. **A** Protocol for AVA in DEN-injected mice fed a high-fat diet. The mice were randomly assigned to NT, NT + HFD, HFD, and HFD + AVA group, and each group had 12 mice. **B**–**C** Avasimibe dramatically suppressed HFD-promoted HCC tumor growth. Representative images of the livers (**B**) and tumor volumes (**C**) from each group of mice were shown; **p < 0.01. **D** Representative H&E-stained images of HCC and adjacent non-tumor tissues from HFD or HFD + AVA group (*n* = 5, scale bar: 50 μm). T: tumor; NT: adjacent non-tumor. The circled position indicated no steatohepatitis area. **E** Representative IHC images of CyclinD1, TIP47, CPT1A, and HMGCL in tumor tissues from HFD-fed mice treated with or without AVA. Data were quantified using ImageJ software (means ± SEM, *n* = 5); *p < 0.05, **p < 0.01.

We further assessed the efficacy of avasimibe in DEN-injected HCC mice on HFD. Avasimibe significantly suppressed HCC growth in HFD-fed mice (Fig. 5A–B). Kaplan*–*Meier survival curves revealed no significant differences (*p* = 0.05), but the HFD + AVA group showed a trend toward better survival rates (Fig. S8). H&E staining showed that avasimibe ameliorated hepatic steatosis in HFD-fed mice (Fig. 5C). Similar to the result observed in vitro (Fig. 4D–H), IHC staining showed that avasimibe down-regulated the expression of cyclin D and TIP47, the LD membrane protein. However, avasimibe promoted the expression of CPT1A and HMGCL, the key enzymes of the FAO and ketogenesis pathway (Fig. 5D). Together, we hypothesize that targeting CPT1A can sensitize HCC cells to SOAT1-targeted therapy.

### Inhibiting CPT1A converts excess FFAs into LDs by SOAT1

**Fig. 6:**
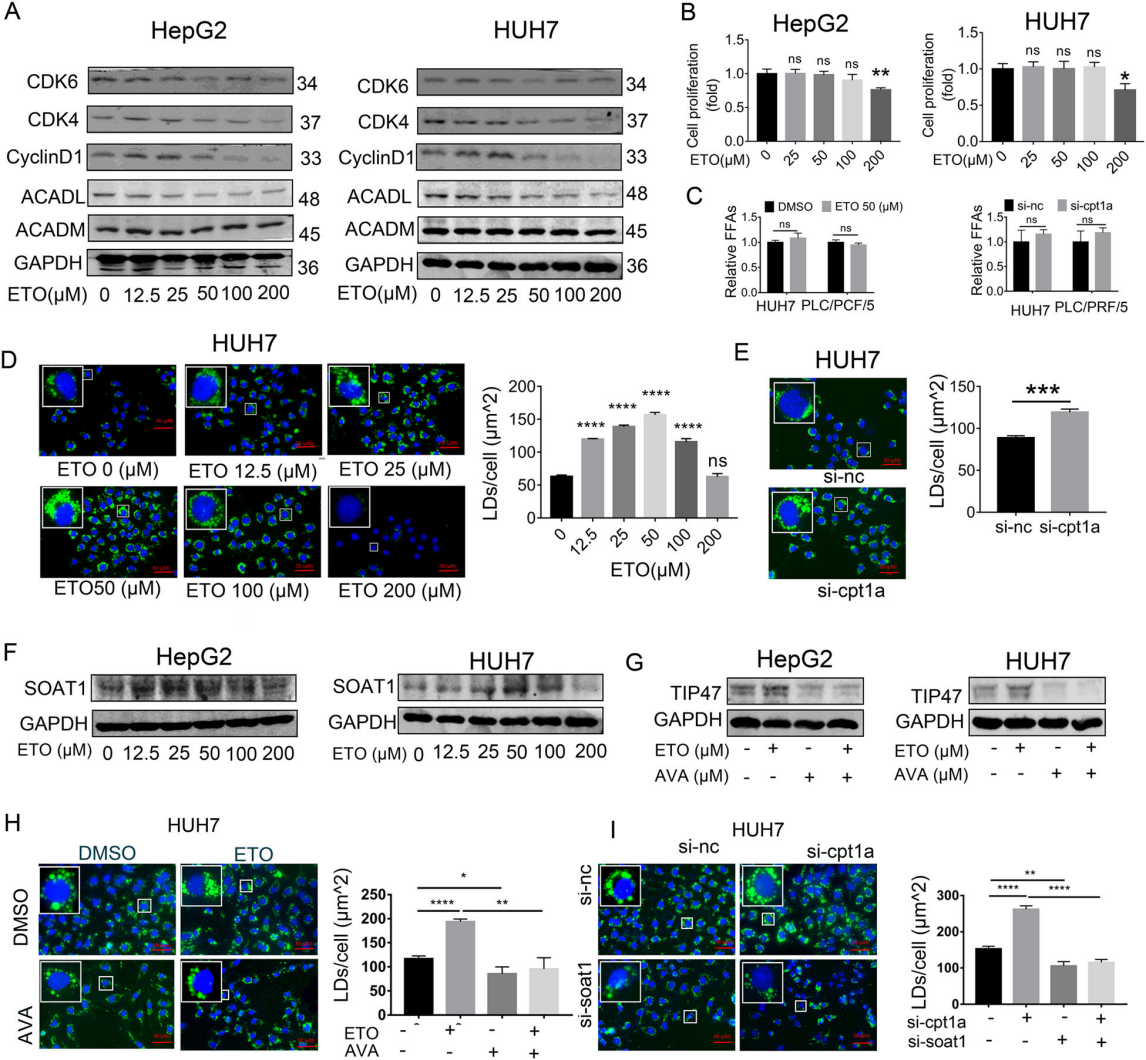
Inhibiting CPT1A converts excessive FFAs into lipid drops by SOAT1. **A** Western blot analysis of CDK4, CDK6, CyclinD1, ACADL, and ACADM in HCC cells treated with ETO at the indicated concentration. **B** CCK8 assay showed that etomoxir (ETO) inhibited cell viability in the HepG2 and HUH7 (means ± SEM, *n* = 3); **p < 0.01, ns, no significance. **C** The level of total free fatty acids in HCC cells treated with CPT1A inhibition (means ± SEM, *n* = 3). ns: no significance. **D**–**E** Fluorescence imaging of LDs stained with BODIPY 493/503 (green) treated with pharmacological (avasimibe, 48 h) or genetic (si-RNA, 72 h) inhibition of SOAT1 in HUH7. Nuclei were stained with DAPI (blue). Scale bar, 50 μm. More than 30 cells were analyzed. Data were quantified using ImageJ software; ***p < 0.001, ****p < 0.0001, ns: no significance. **F** Western blot analysis of SOAT1 in HepG2 and HUH7 treated with ETO at the indicated concentration for 48 h. **G**–**I** Cellular LDs level in HCC cells treated with ETO plus AVA. More than 30 cells were analyzed.; *p < 0.05, ***p < 0.001, ****p < 0.0001.

Previous research uncovered that CPT1A-dependent FAO plays an important role in the cell cycle progression of CRC cells^29^ and targeting CPT1A induced a cell cycle arrest^30^. Our study found etomoxir indeed blocking FAO pathway by decreasing the expression of long-chain-acyl-CoA dehydrogenase (ACADL), which is another enzyme of fatty acid oxidation (FAO). Moreover, the high concentration of etomoxir can reduce cell cycle-related proteins such as CDK4 and cyclinD1 (Fig. 6A). Consistent with the previous studies^31^, the CCK8 assay indicated etomoxir slows the proliferation of HCC cells (Fig. 6B). However, the IC50 of etomoxir exceeded 150 μM, which was far from satisfaction. The potential mechanism of the undesirable efficacy is still poorly understood, and a sensitizer that enhances the utility of etomoxir is urgently needed. We speculate inhibition CPT1A can increase intracellular FFAs by blocking their oxidization. But no changes were observed in FFAs levels after CPT1A inhibition (Fig. 6C). In our study, we further found targeting CPT1A increased the expression of SOAT1 and the number of LDs (stained by BODIPY 493/503) (Fig. 6D–F). Thereby, we hypothesize that inhibiting CPT1A forces FFAs to form LDs via SOAT1. To further elucidate the idea, we performed rescue experiments. The result showed that targeting SOAT1 partly reverted the level of LDs promoted by inhibiting CPT1A (Fig. 6G–I). Thereby, Targeting CPT1A converted FFAs to form LDs via SOAT1.

In summary, SOAT1 and CPT1A formed a double-negative feedback loop to keep lipid homeostasis in HCC. As an organism can alter the metabolic pathway to circumvent the single drug-caused cytotoxicity, simultaneously targeting SOAT1 and CPT1A might achieve better efficacy in HCC.

### Combined avasimibe and etomoxir enhances the efficacy by disrupting lipid homeostasis in HCC in vitro and in vivo

**Fig. 7:**
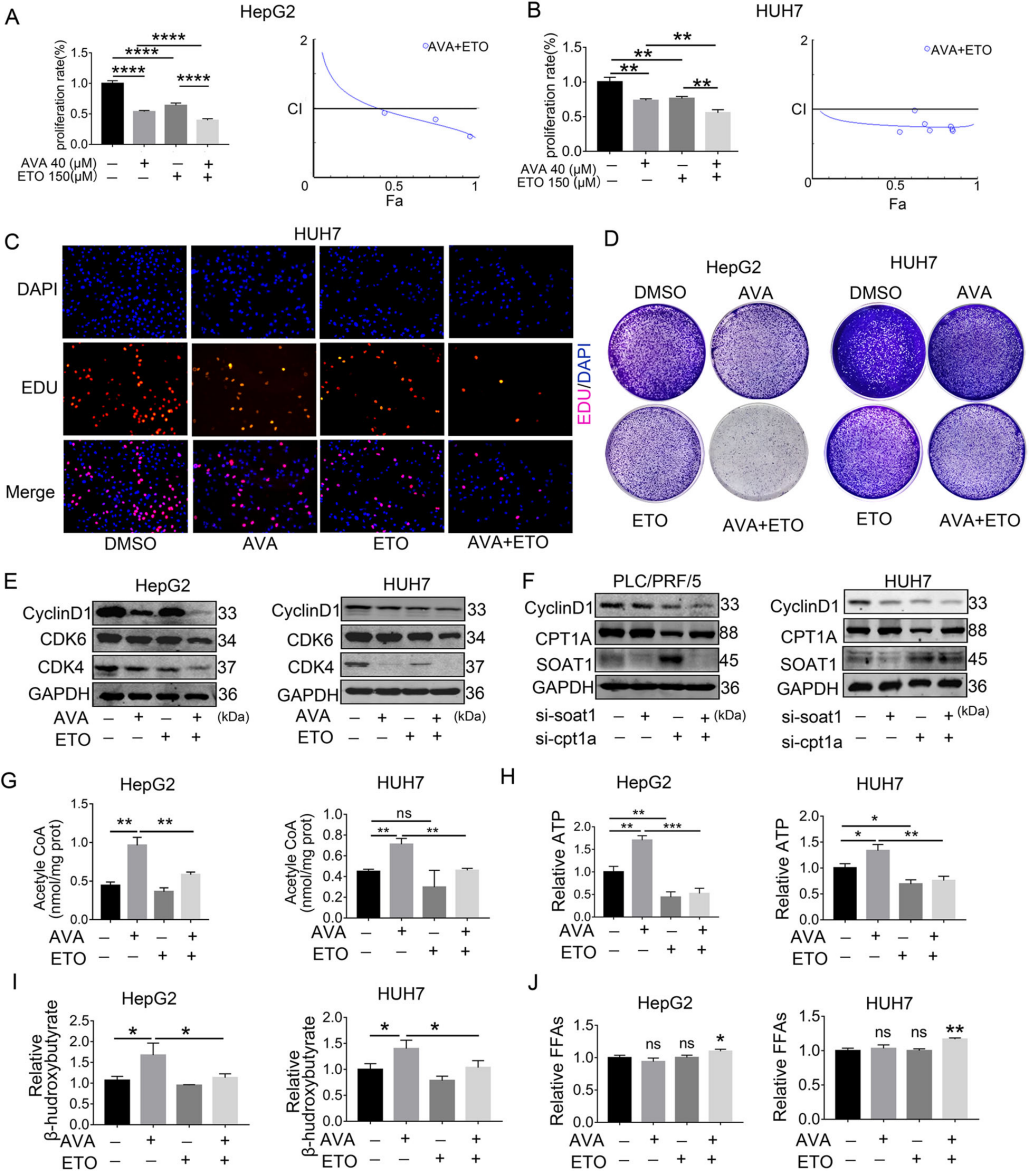
Combined avasimibe and etomoxir enhance the efficacy by disrupting lipid homeostasis in HCC in vitro. **A**–**B** CCK8 assay showed that combined avasimibe and etomoxir enhanced the efficacy in the HepG2 and HUH7 (means ± SEM, *n* = 3). Chou and Talalay analyzed the combination indices (CI) of combined AVA and ETO for 48 h in HepG2 and HUH7. The straight line at CI = 1 represents the additive effects of both drugs. Fraction effect (FA): growth inhibition rate. **C** Representative images of the EdU staining assay in HUH7 cells treated with AVA, ETO, or both. **D** The colony formation assay of HepG2 and HUH7 cells cultured with 2 μM AVA and/or 25 μM ETO for 14 days. **E** Western blot analysis of CDK4, CDK6, and Cyclin D1 in HepG2 and HUH7 cells treated with 20 μM AVA and/or 150 μM ETO. **F** Western blot analysis of CyclinD1, CPT1A, and SOAT1 after transfection with si-SOAT1and/or si-CPT1A in PLC/PCF/5 and HUH7. **G**–**I** Levels of total acetyl-CoA, ATP and β-hydroxybutyrate in HCC cells treated with AVA (5 μM) and/or ETO (50 μM) (mean ± SEM, *n* = 3); *p < 0.05, **p < 0.01, ***p < 0.001; ns: no significance. **J** Levels of intrahepatic FFAs in HepG2 and HUH7 treated with AVA (5 μM) and/or ETO (50 μM) (mean ± SEM, *n* = 3); *p < 0.05.

The combination of avasimibe and etomoxir was more effective in restraining cell proliferation of HCC in vitro. Data from the CCK8 assay was analyzed using Calcusyn software. The result revealed the combination index (CI) was below 1.0, which indicated the interactions between avasimibe and etomoxir were synergistic (Fig. 7A–B). In addition, EdU assays and colony formation assay showed similar results as the CCK8 assay (Fig. 7C–D). Figure S9 showed the quantification results of EdU assays. The Western blot analysis also showed the combination group inhibited CDK4, CDK6, and cyclinD1 more effectively than the single group (Fig. 7E). The main reason was SOAT1 and CPT1A formed a double-negative feedback loop (Fig. 7F). Besides, avasimibe plus etomoxir can partly rescue the rise of acetyl-CoA, ATP, and β-hydroxybutyric acid mediated by avasimibe in HCC cell lines (Fig. 7G–I). Again, these results proved that avasimibe initiated FAO activity through CPT1A enhancement. Moreover, compared with each monotherapy, the combination treatment slightly elevated FFAs in HCC cells (Fig. 7J). Excessive FFAs can cause cytotoxicity, leading to cell damage. To test the idea, we examined cell proliferation in HCC cell lines treated with palmitic acid in the dose range of 0–200 μM. High-dose palmitic acid markedly induced cell death, while low level benefited cell proliferation (Fig. S10). A previous study has demonstrated that low-level FFAs below the physiological level induced a beneficial effect on mitochondrial function and liver health, but high-level FFAs above the physiological level induced lipotoxicity^32^. Collectively, the combination of avasimibe and etomoxir enhanced cytotoxic effects by FFAs overload in HCC in vitro.

**Fig. 8:**
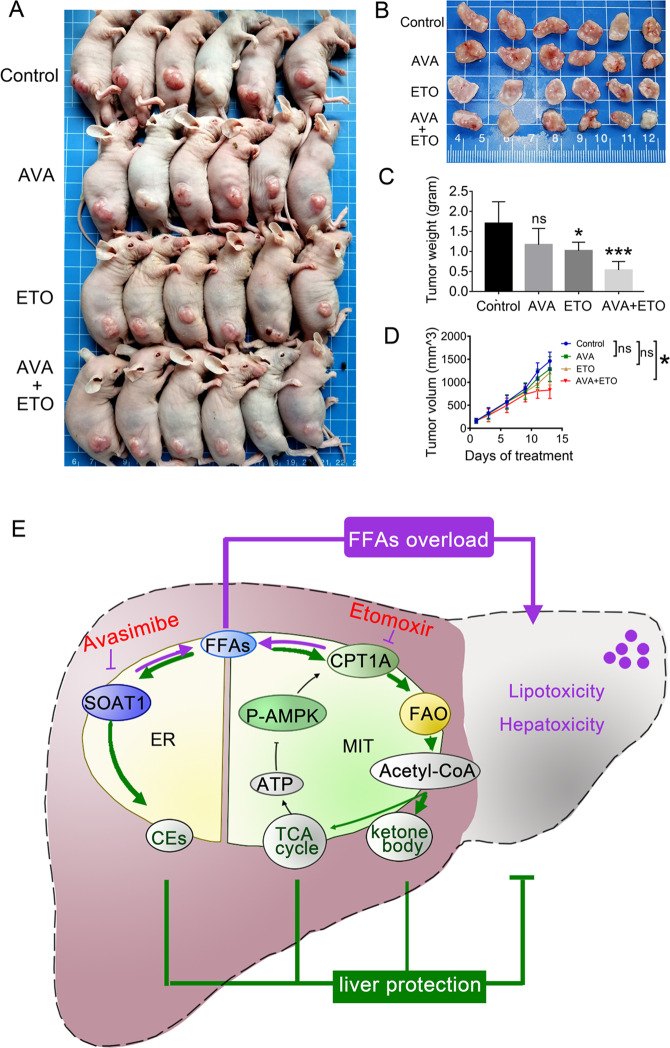
Avasimibe and etomoxir augmented anti-cancer activity in HCC xenograft mouse model. **A**–**B** A combination of avasimibe and etomoxir effectively inhibited tumor growth. **C** Tumor weights on the final day. **D** Tumor growth curves in mouse xenograft models treated with avasimibe (15 mg kg^−1^ day^−1^), etomoxir (10 mg kg^−1^ day^−1^), or both. Circle and triangle denote the average volume of tumors (mean ± SEM, *n* = 8). **E** A mechanism graph illustrating how SOAT1 and CPT1A regulate lipid homeostasis to avoid lipotoxicity in HCC. SOAT1 and CPT1A balance excess FFAs by converting them in ER to form lipid drops and in mitochondria for oxidation. If one of the two fatty acids degradation pathways is blocked, another pathway is activated to maintain lipid homeostasis. Simultaneously targeting SOAT1 and CPT1A by the small-molecule inhibitors avasimibe and etomoxir had synergistic anticancer efficacy by disrupting lipid homeostasis. ER endoplasmic reticulum, MIT mitochondria.

To test in vivo the combinatorial therapeutic efficacy, we established a xenograft model by nude mice with HepG2 cells. The mice were intraperitoneally injected with avasimibe (15 mg/kg/day) and/or etomoxir (10 mg/kg/day) for 14 days. The monotherapy slightly reduced the tumor volume and weight, but their combination significantly suppressed tumor growth (Fig. 8A–D). During the administration, no apparent weight loss or abnormal behavior was observed in the nude mice. Together, combined avasimibe and etomoxir augmented anti-cancer activity in vivo.

## Discussion

Reprogramming of lipid metabolism has been reported in various cancers^33^. But how HCC cells regulate lipid homeostasis to prevent lipotoxicity has rarely been investigated. Previous studies showed upregulation of CPT1A is essential for the tumor-promoting effect^21^. Herein, we found HFD elevated the expression of CPT1A. Thereby, we deduced HFD can accelerate HCC progression via CPT1A. Another recent study demonstrated hepatic steatosis accelerated HCC progression^34^. We further revealed HFD-promoted hepatic steatosis and HCC progression through SOAT1. These findings imply that overexpression of SOAT1 and CPT1A may enable HCC cells to adapt to the lipid-rich environment and thus avoid lipotoxicity.

Since 2019, SOAT1 has become a hot therapeutic target for cancers^35–37^. But fatty acids are regulated by a complex mechanism. Targeting SOAT1 might contribute to metabolic alteration, which offset the efficacy of SOAT1 inhibition in HCC. Our data showed that SOAT1 inhibition significantly upregulated the CPT1A protein, which can facilitate the entry of excess FAs into the mitochondria for oxidation, leading to a remarkable rise in acetyl-CoA production. Although a previous work reveals avasimibe didn’t affect CPT1A mRNA levels in glioblastoma using RNA-sequencing analyses^24^, the reason might be the tissue specificity. The CPT1 family comprises three subtypes, CPT1A (liver type), CPT1B (muscle type), and CPT1C (brain type). Similar to our result of bioinformatics analysis, CPT1A is strongly expressed in HCC, but lower in glioblastoma (Fig. 3B). We found avasimibe amplified ketogenesis via a positive-feedback loop. FAO-derived acetyl-CoA is either converted to ketone bodies through ketogenesis or further oxidize to ATP through the TCA cycle. Avasimibe causes sustained activation of ketogenesis. Enhancement of ketogenesis condenses acetyl-CoA into ketone bodies, which accordingly decreases the flux of acetyl-CoA into the TCA cycle^38^. The reduced ATP stimulates AMPK*/*ACC axis, which in return activates the FAO and ketogenesis pathway. Ketone bodies generated from FAO act as an alternative source of energy when glucose supply is depleted and promote tumor progression. Based on the evidence above, we can safely conclude that avasimibe upregulates CPT1A expression and CPT1A inhibition can sensitize HCC cells to avasimibe treatment.

Etomoxir impacts energy metabolism and suppresses tumor growth in prostate cancer and leukemia, which agrees with our result^39,40^. However, the IC50 of etomoxir exceeded 150 μM, which was far from satisfaction. A sensitizer that enhances the utility of etomoxir is urgently needed. A previous study shows that CPT1A inhibition promotes the formation of lipid droplets^39^. We further verified that etomoxir forces fatty acids to form LDs via SOAT1. Thereby, SOAT1 inhibition can sensitize HCC cells to CPT1A-targeted treatment.

Maintenance of cellular lipid homeostasis is essential for cell survival and disruptions to homeostasis lead to diseases^41–43^. A previous study has demonstrated that low-level FFAs below the physiological level induced a beneficial effect on mitochondrial function and liver welfare, but the high-level FFAs above the physiological level induced lipotoxicity^32^. Our results indicated that combined avasimibe and etomoxir elevated FFAs in HCC cells compared with each monotherapy. Moreover, SOAT1-mediated CEs synthesis and CPT1A-mediated FAO formed alternative pathways to balance intracellular FFAs levels, combined avasimibe and etomoxir had greater efficacy than monotherapy by disrupting lipid homeostasis.

Avasimibe and etomoxir have been tested in clinical trials for the treatment of metabolic disease and have good human safety^44,45^. Thereby, our work provides a strong basis for the translation of combined avasmibe and etomoxir into clinical trials for treating HCC, especially for steatohepatitis HCC.

## Materials and methods

### Chemicals and antibodies

Triton X-100, PEG300, and Tween 80 were purchased from Sigma-Aldrich. Bodipy 493/503 was purchased from Thermo Fisher Scientific. Filipin III was purchased from Santa Cruz. Etomoxir, avasimibe, palmitic acid, and oleic acid were purchased from Tsbiochem in China. Diethylnitrosamine was purchased from Tokyo Chemical Industry in Japan. 60% HFD was purchased from Moldiets, Biopike in China. Rabbit anti-CDK4(ab108357, 1:1000 for WB), rabbit anti-CDK6 (b124821, 1:1000 for WB) were purchased from Abcam (Cambridge, UK). Rabbit anti- SOAT1 (A6311, 1:1000 for WB) was purchased from Abclonal. Rabbit anti-SOAT1 (NB400-141, 1:1000 for IHC*)* was purchased from Novus. Rabbit anti-CyclinD1 (26939-1-AP, 1:1000 for WB, 1:100 for IHC), Anti-CPT1A (15184-1-AP, WB: 1/1000; IHC:1/100), Anti-Perilipin 3/TIP47 (10694-1-AP, WB: 1/1000; IHC 1/100), Anti-ACADM (55210-1-AP, WB: 1/500), Anti-ACADL (17526-1-AP, WB: 1/500), Anti-GAPDH were purchased from Proteintech.

### Hepatic tumor in situ

Animal experiments were carried out according to the protocol approved by the Institutional Committee of Animal Care and Use of UESTC & Sichuan Provincial Hospital (China). Male C57BL/6J mice, three weeks old, were purchased from Beijing HFK Bioscience. For liver carcinogenesis, the mice were intraperitoneally injected with either diethylnitrosamine (DEN, 80 mg/kg) or saline once a week for five weeks. The DEN-injected mice on a ND were named ND group. The DEN-injected mice on a 60% HFD were named HFD group. After 12 weeks of HFD, obese mice were gavaged with the suspension of avasimibe (35 mg/kg, three times/week) was named HFD + AVA. All mice were housed for 12 months before euthanized. Since the tumors were spread on the liver, all the tumors of each mouse were collected in a measuring cylinder, and filled up with PBS to the final volume. The tumor volume = final volume−PBS volume.

### cBioPortal

The cBio Cancer Genomics Portal (http://cbioportal.org)^46^, as an online analysis tool, is mainly used for the exploration of multidimensional cancer genomics data sets whose resource comes from more than 5000 tumor samples of 20 cancer studies.

### UALCAN

UALCAN (http://ualcan.path.uab.edu)^47^ is an online tool whose resource mainly comes from the level 3 RNA-seq and clinical data of 31 cancer types from the TCGA database. This tool is commonly used when analyzing gene expression profiles and relationships between mRNA expression and clinical characteristics.

### LinkedOmics

The LinkedOmics database (http://www.linkedomics.org/login.php) is an open-access online biometrics platform whose resource comes from 11,158 patients from the TCGA^48^. In this study, we analyzed genes differentially expressed in correlation with SOAT1 or CPT1A in the TCGA HCC cohort (*n* = 371), committed to finding and assessing the correlation between genes by Pearson’s correlation coefficient.

### Gene set enrichment analysis (GSEA)

GSEA (https://www.broadlnstitute.org/gsea/) was performed using the Molecular Signatures Database (MSigDB) version 6.0^49^. In the text, genes in each gene set are available at MSigDB: c2 KEGG, c5 CC, c5 MF.

### Immunohistochemistry

The tissues obtained from DEN-induced HCC mice were fixed overnight in 4% paraformaldehyde, embedded in paraffin, and cut into 5 μm sections. The sections were subjected to haematoxylin and eosin staining, as well as immunohistochemical (IHC) staining, following standard protocols. IHC quantification Immunohistochemical reactions were quantified using Image J software.

### Cell culture

HepG2, HUH7, and PLC/PRF/5 cells were purchased from ATCC (Manassas, VA, USA). Cells were cultured in Dulbecco’s modified Eagle medium, supplemented with 10% fetal bovine serum (FBS; all from Gibco/Invitrogen, USA).

### Cell viability and cell proliferation assessment

For cell proliferation assays, cells were seeded in 96-well plates and allowed to attach overnight. After transfection or treatment with the indicated drugs, relative cell growth was measured using the Cell Counting Kit-8 (Dojingdo, Kumamoto, Japan). For colony formation assays, cells were seeded into a 35 mm dish and cultured in the Dulbecco’s modified Eagle medium with 10% FBS overnight. Cells were then treated with avasimibe or/and etomoxir as indicated in complete media for seven days. Growth media with or without the drug was replaced every two days. The remaining cells were fixed with methanol (1%) and formaldehyde (1%), stained with 0.5% crystal violet, and photographed using a digital scanner.

### EdU assay

Cell proliferation was also determined by the 5-ethynyl-2′-deoxyuridine (EdU) assay using a Cell-LightTM EdU Apollo®488 In Vitro Imaging Kit (Guangzhou RiboBio Co., LTD). The cells were then visualized under fluorescence microscopy (20 × 10). At last, the ratio of EdU-stained cells (with red fluorescence) to Hoechst-stained cells (with blue fluorescence) was calculated.

### Western blot

Cells were washed twice with PBS and lysed in RIPA buffer ((Trisbase 50 nM, NaCl 150 mM, NP-40 1%, sodium deoxycholate 0.25%, and EDTA 1 mM) containing a cocktail of protease inhibitors and phosphatase inhibitors (Calbiochem, Darmstadt, Germany). Equal amounts of protein sample (30–50 μg) were separated by 12% SDS-PAGE and transferred to nitrocellulose membrane (Millipore, Bedford, MA, USA) using the BioRad wet transfer system.

### Lipid droplets staining and quantification

Cells were fixed with 4% paraformaldehyde and permeabilized with 1% Triton X-100 for 15 min. After washing with cold phosphate-buffered saline (PBS), cells were stained with BODIPY 493/503 (0.5 μM, Thermo Scientific) for 30 min and then stained with 5 μg/ml DAPI for 5 min (Invitrogen) at room temperature. The LDs were detected using a fluorescence microscope (Eclipse 80i, Nikon, Japan) at ×200 magnifications. More than 30 cells were analyzed, and LDs numbers were quantified with the ImageJ software.

### Free cholesterol assessment

Filipin-III was used to visualize free cholesterol in cells. Cells were fixed with 4% paraformaldehyde and permeabilized with 1% Triton X-100 for 15 min. After washing with cold PBS, cells were firstly incubated with 100 μg/mL Filipin III for 2 h at room temperature. Fluorescence pictures were detected using a fluorescence microscope (Eclipse 80i, Nikon) at ×200 magnifications and analyzed using ImageJ software.

### FFAs assessment

FFA was measured in HCC cell lines by a FFA assay kit (Solarbio, China) according to the protocols provided by the manufacturer.

### ATP generation

The cellular ATP concentrations were determined by an ATP detection kit (Beyotime, China). Cells grown in six-well plates were washed twice with PBS, add 200 μL lysis buffer from the ATP detection kit, and then ultrasonicated. The lysate was centrifuged at 12,000 × *g* for 5 min at 4 °C. The supernatant was transferred to a new 1.5 mL tube for an ATP test. The relative ATP level was calculated according to the following formula: relative ATP level = ATP value/protein value. The protein value of the sample was measured by a BCA protein assay kit (Pierce, Thermo Scientific).

### Acetyl-CoA assessment

Acetyl-CoA was measured in HCC cell lines by acetyl-CoA assay kit (Solarbio, China) according to the protocols provided by the manufacturer. Cells (1 × 10^4^) were trypsinized, lysed with lysis buffer (the reagent 1 of the commercial kit.). After centrifuging at 13,000 × *g* for 10 min, supernatants were collected and used for acetyl-CoA measurements. Relative acetyl-CoA level (nmol/mg prot) = (1640 × ∆A + 0.012) ÷Cpr; ∆A = A2 − A1, A1: the absorbance value at 340 nm after 20 s, A2: the absorbance value at 340 nm after 80 s. Cpr: the protein value. The protein contents were determined using the BCA Protein Assay Kit.

### Ketone body assessment

β-hydroxybutyrate was measured in HCC cell lines by β-hydroxybutyrate assay kit (Mlbio, China) according to the protocols provided by the manufacturer.

### Transfection

Lipofectamine 2000 (Invitrogen) was used for transfection according to the manufacturer’s instructions. After 72 h, the transfected cells were used for subsequent experiments. The effect of RNA interference was maintained at least for a week. Si-RNAs of SOAT1, CPT1A, and negative control were obtained from GenePharma (Shanghai, China). Sequences of the si-RNA were as follows:

si-SOAT1: 5′-3′GCUCGUGUUCUGGUCCUAUTT-3′;

si-CPT1A#1: 5′--3′GAAGCUCUUAGACAAAUCTT,

si-CPT1A#2: 5′-3′GCCUUUACGUGGUGUCUATT,

si-CPT1A#3: 5′-3′UCAAUGGACAGCUACGCCTT.

To improve the knockdown efficiency of CPT1A, the three different si-RNA were mixed in our study.

### In vivo xenograft experiments

Male BALB/c nude mice, 5–6 weeks old, were obtained from Beijing HFK Bioscience. Mice were subcutaneously injected with HepG2 (5. 0 × 10^6^ cells). A week after implantation, mice with a tumor size of ~180 mm^3^ were subsequently assigned to four groups (*n* = 8). Avasimibe or etomoxir were dissolved in saline with 5% DMSO, 5% Tween 80, and 20% PEG300. Mice were daily intraperitoneal injected with vehicle (saline with 5% DMSO, 5% Tween 80, and 20% PEG300), avasimibe (15 mg/kg), etomoxir (10 mg/kg/d), or combined drugs for 28 days. Tumor diameters were serially measured with a digital caliper (Proinsa, Vitoria, Spain) every three days, and tumor volumes were calculated using the following formula: *V* = (L*W2)/2, where *L* and *W* represent length and width. All mice experiments were performed under the institute guidelines and were approved by the animal ethics committee of the China Institute of Science.

### Statistical analysis

The student’s *t*-test (two-tailed) was used for differences between groups in cytotoxicity assays and gene expression analysis by GraphPad Prism 5. The log-rank test was used to compare the survival plot of each group. Statistical significance was defined at *p < 0. 05, **p < 0. 01, and ***p < 0. 001.

The synergistic inhibition effect on the viability of HCC cells between avasimibe and etomoxir was analyzed by the Calcusyn software (Biosoft, Cambridge, England), which uses the CI method of Chou and Talalay^50^, based on the multiple drug effect equation. The constant ratio combination design was applied to assess the effect of both drugs in combination, in which dose-response curves were determined. The advantage of this method is the automatic construction of a fraction affected-CI table, graph, and calculation of dose reduction indices by the software. In this mathematical model, CI value <1 indicates synergistic effect; CI value of 1 indicates additive effect; CI value >1 indicates antagonistic effect. All of the experiments were performed independently at least three times.

## Availability of data and materials

The datasets supporting the findings of this study are included in the article.

## Supplementary information

Figure S1

Figure S2

Figure S3

Figure S4

Figure S5

Figure S6

Figure S7

Figure S8

Figure S9

Figure S10

Supplementary figure legends
