# Supplementary figures and images for: Simultaneously targeting SOAT1 and CPT1A ameliorates hepatocellular carcinoma by disrupting lipid homeostasis
Source: Cell Death Discov. 2021 May 29;7:125. doi: 10.1038/s41420-021-00504-1 (PMC8164629; doi:10.1038/s41420-021-00504-1)

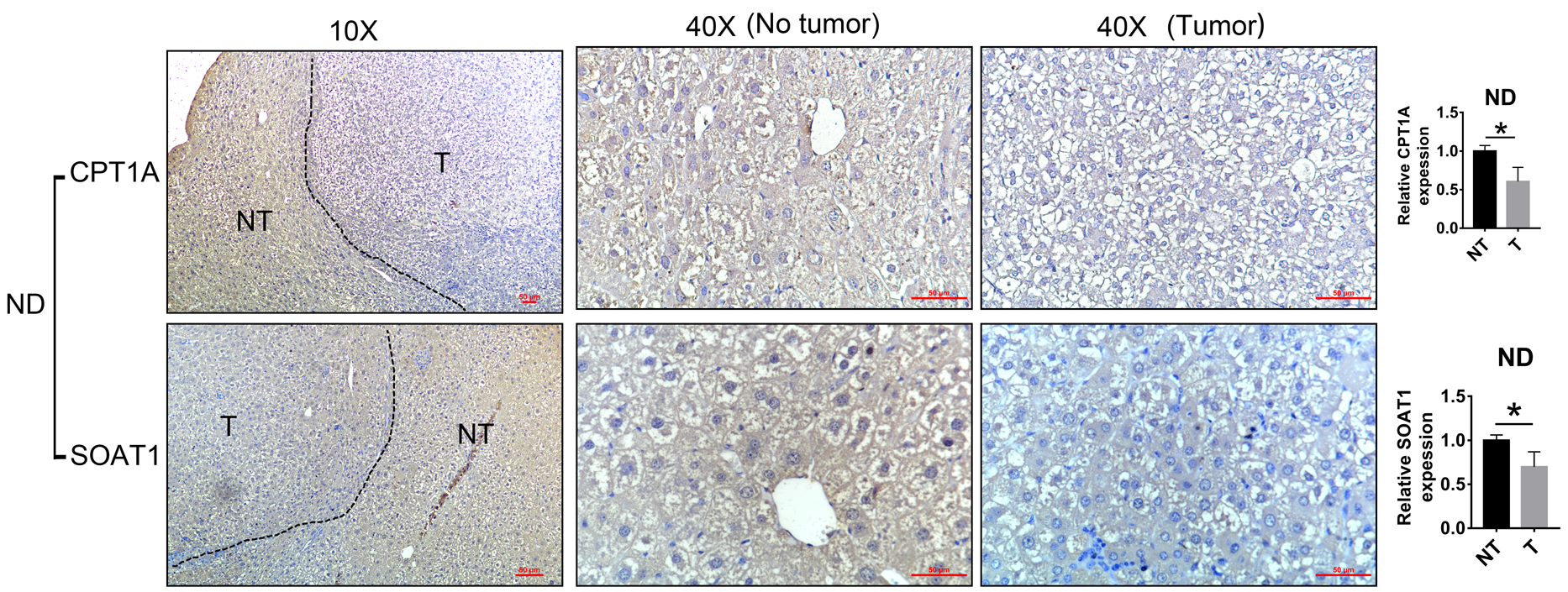

Supplement: Supplementary file 1 — Figure S1 [file 41420_2021_504_MOESM1_ESM.tif]

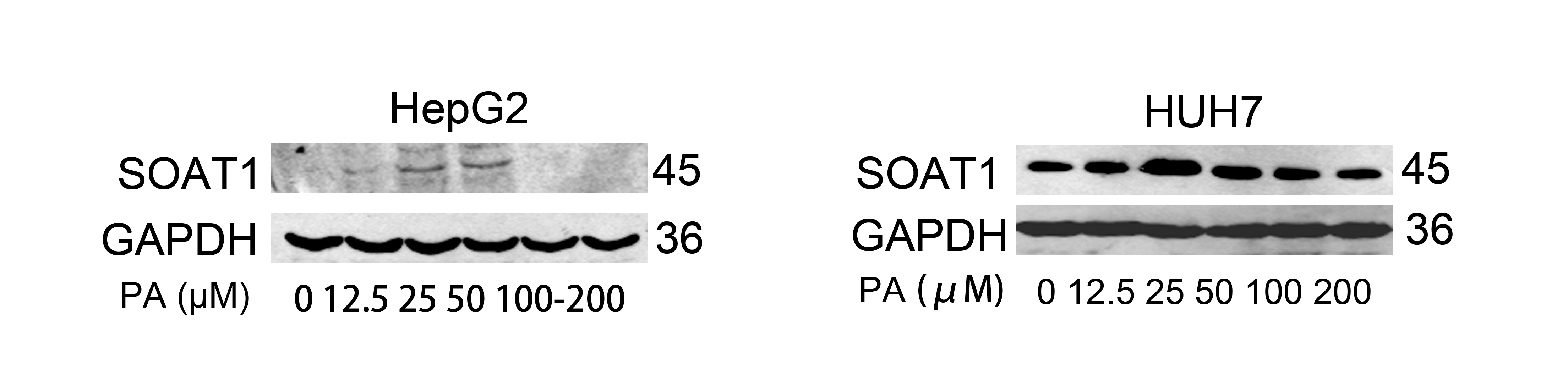

Supplement: Supplementary file 2 — Figure S2 [file 41420_2021_504_MOESM2_ESM.tif]

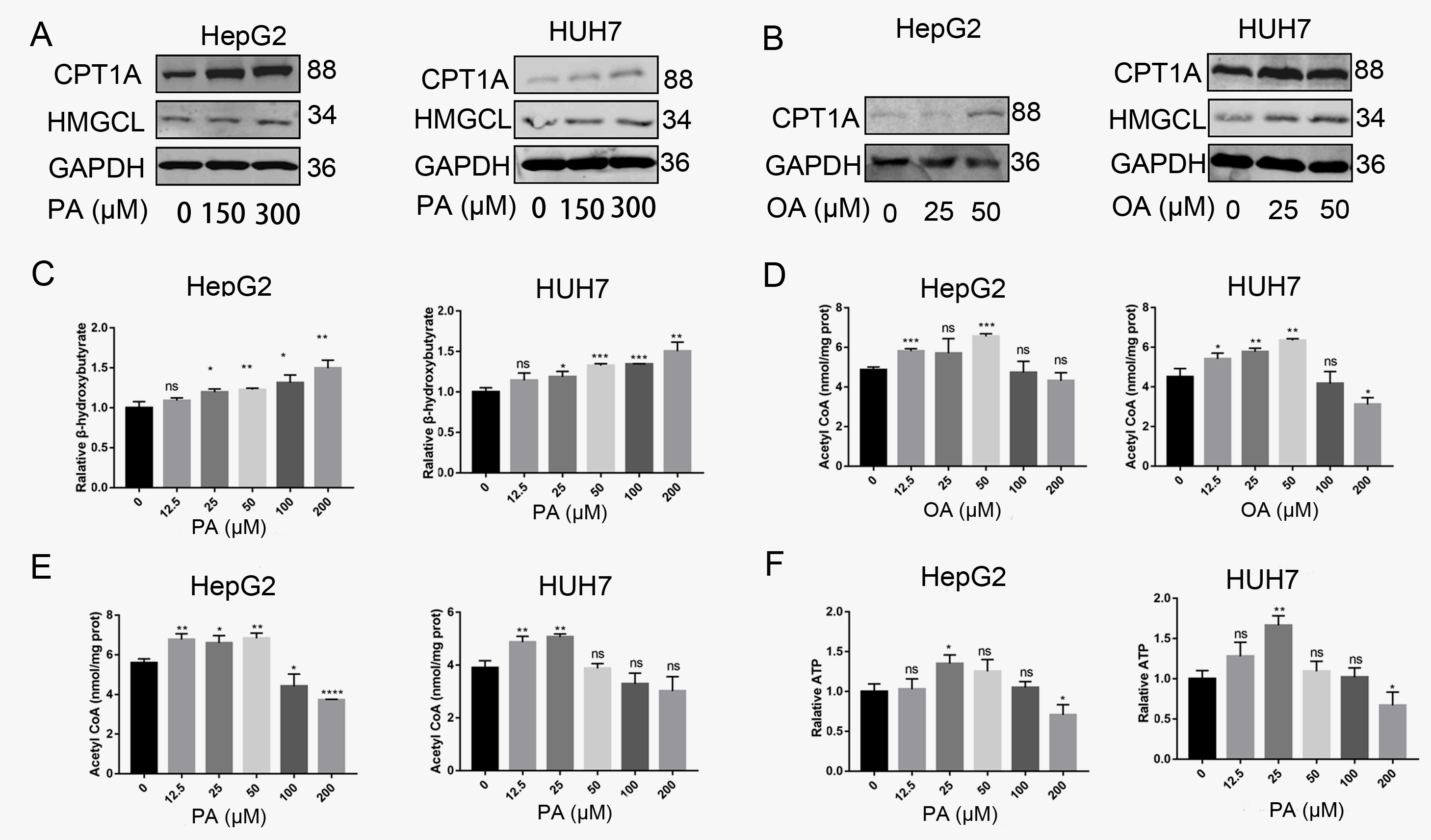

Supplement: Supplementary file 3 — Figure S3 [file 41420_2021_504_MOESM3_ESM.tif]

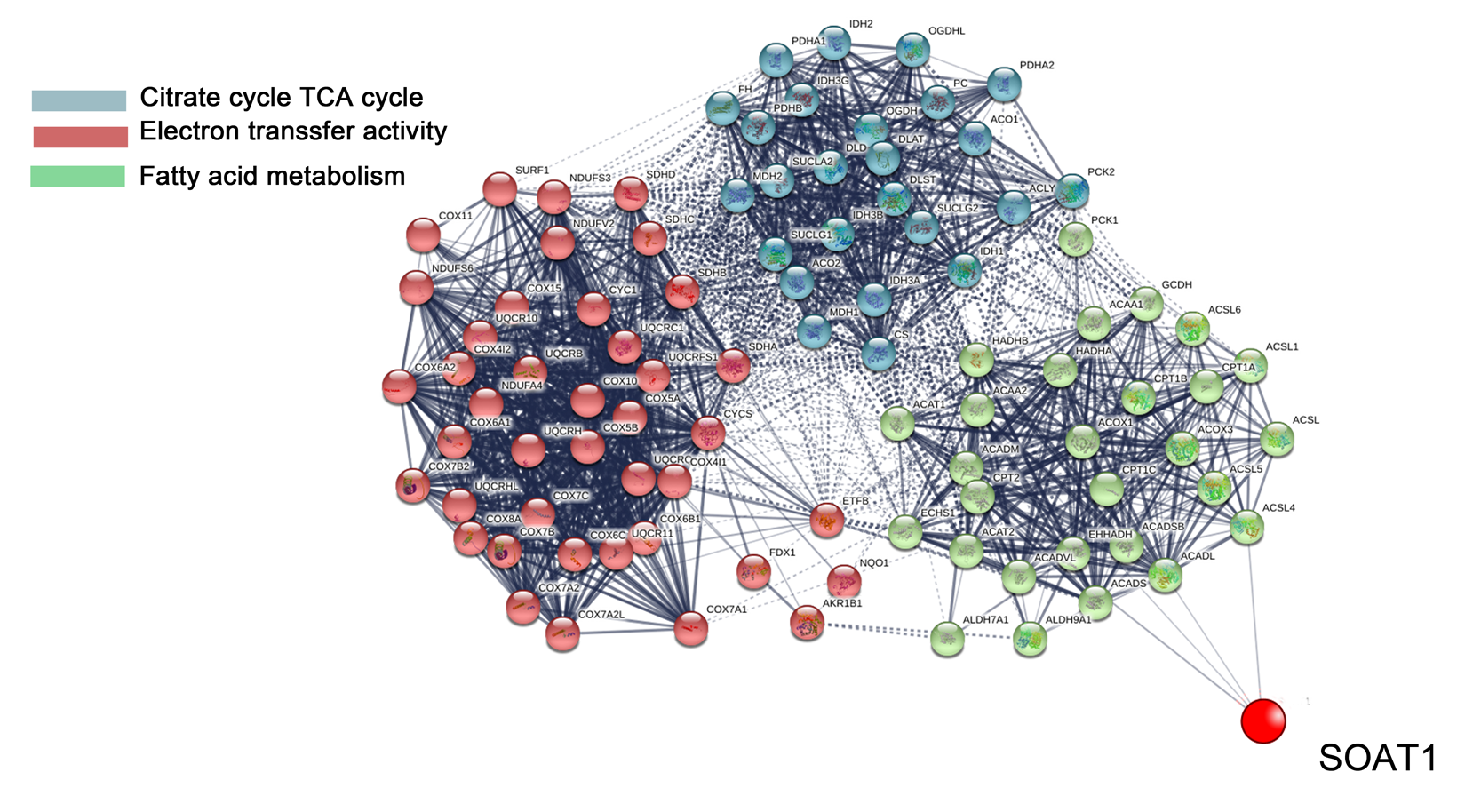

Supplement: Supplementary file 4 — Figure S4 [file 41420_2021_504_MOESM4_ESM.tif]

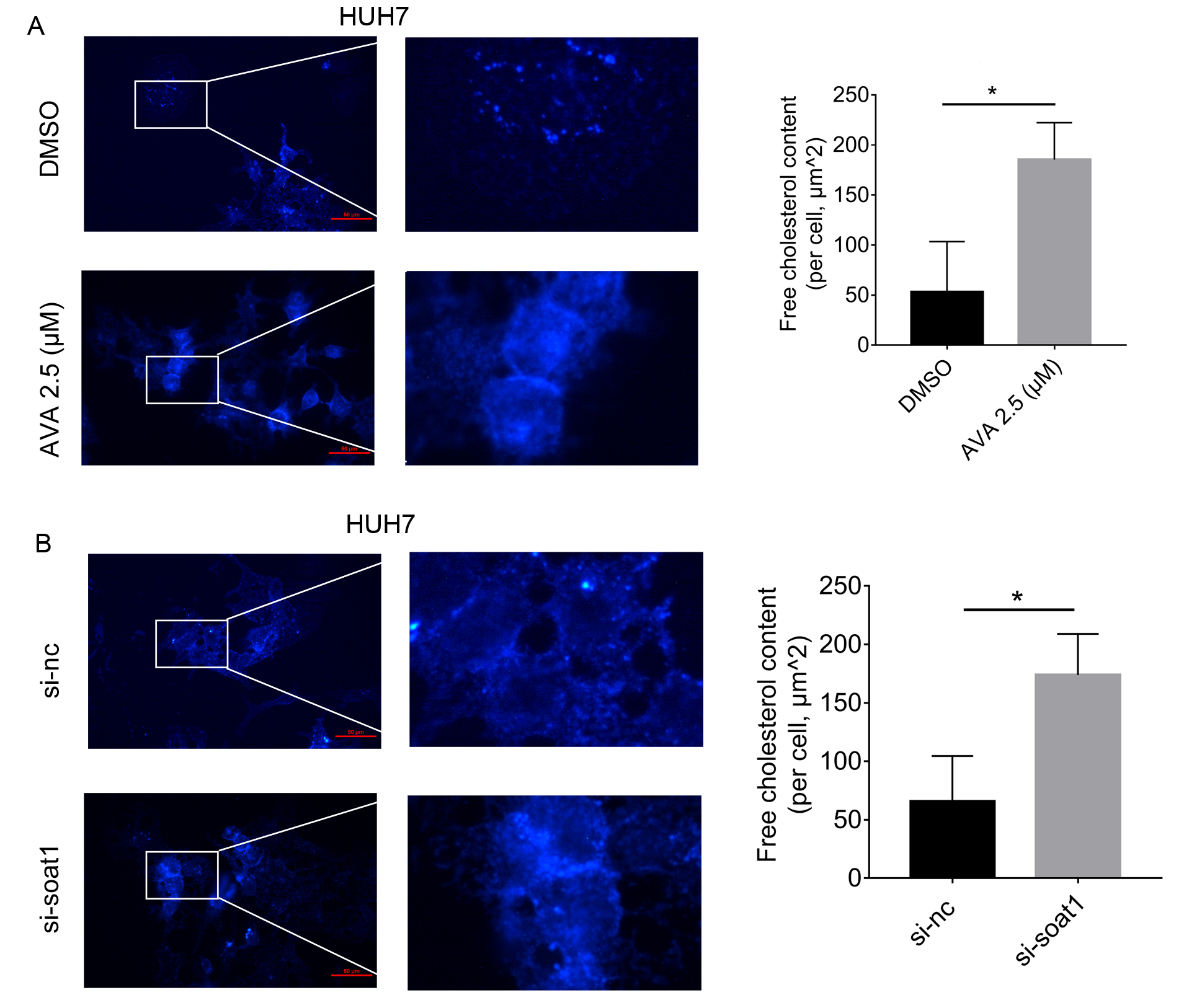

Supplement: Supplementary file 5 — Figure S5 [file 41420_2021_504_MOESM5_ESM.tif]

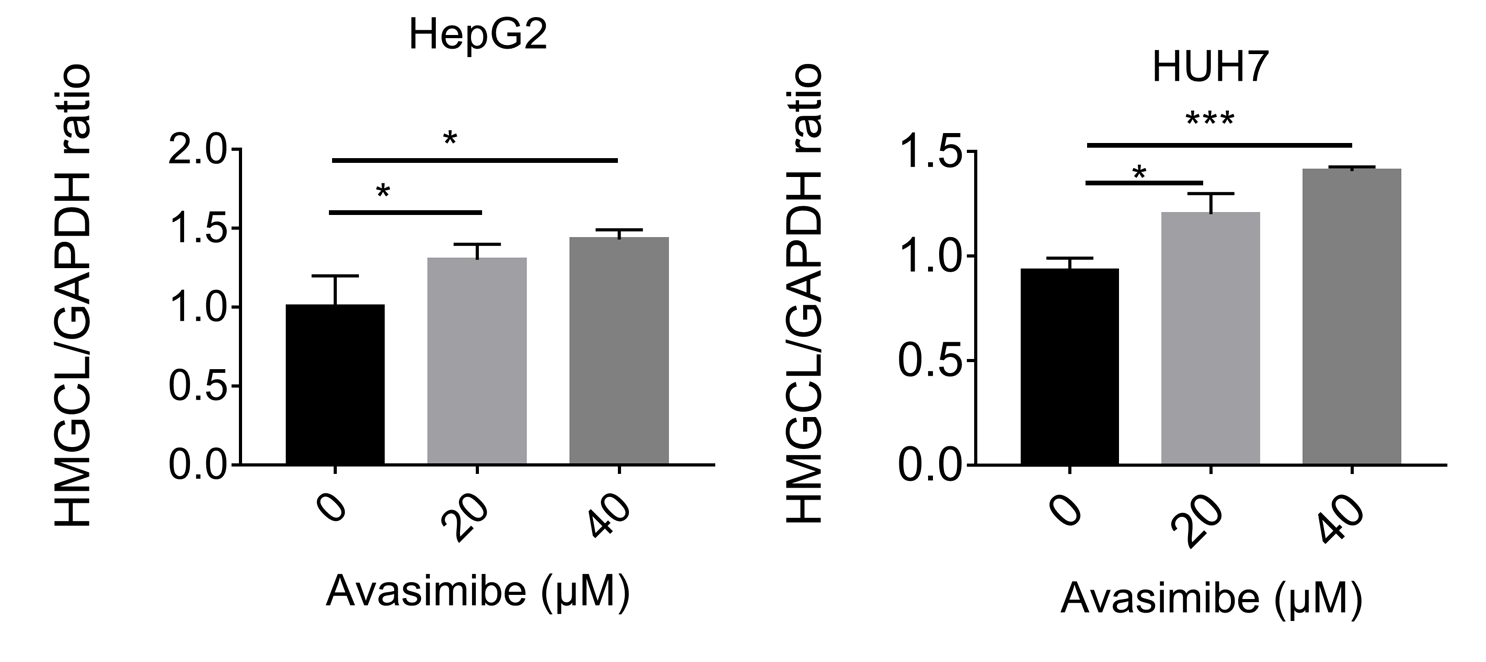

Supplement: Supplementary file 6 — Figure S6 [file 41420_2021_504_MOESM6_ESM.tif]

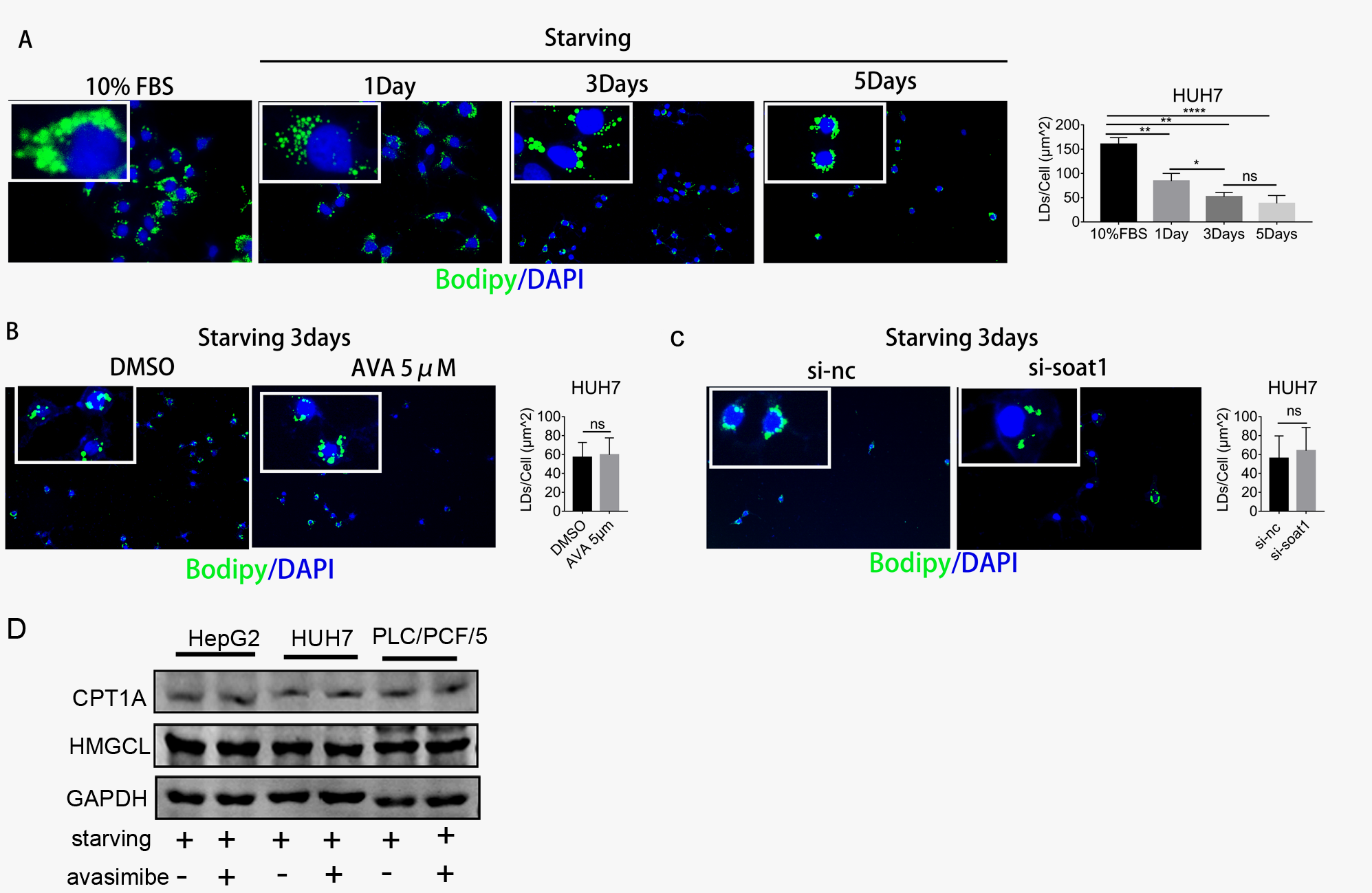

Supplement: Supplementary file 7 — Figure S7 [file 41420_2021_504_MOESM7_ESM.tif]

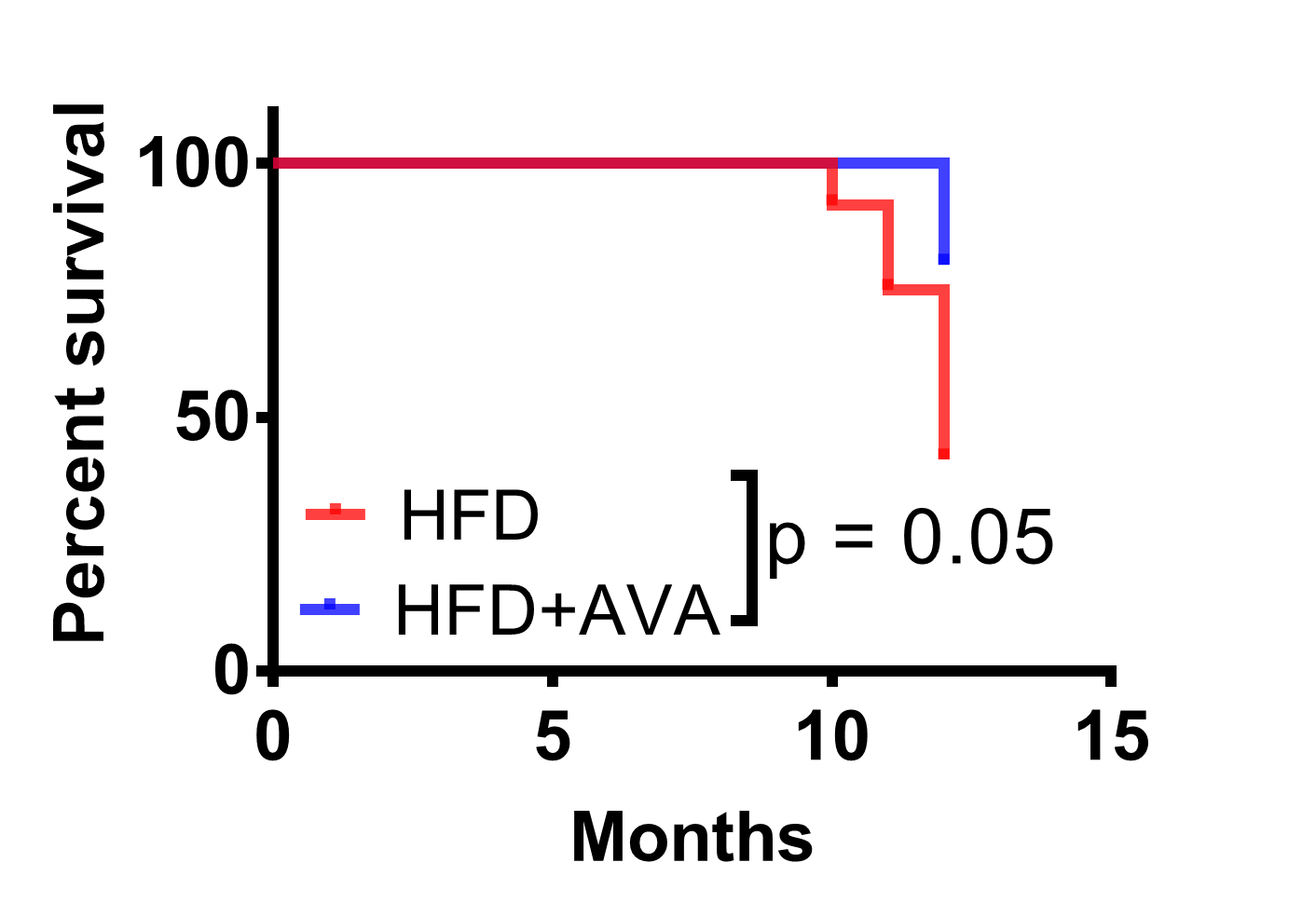

Supplement: Supplementary file 8 — Figure S8 [file 41420_2021_504_MOESM8_ESM.tif]

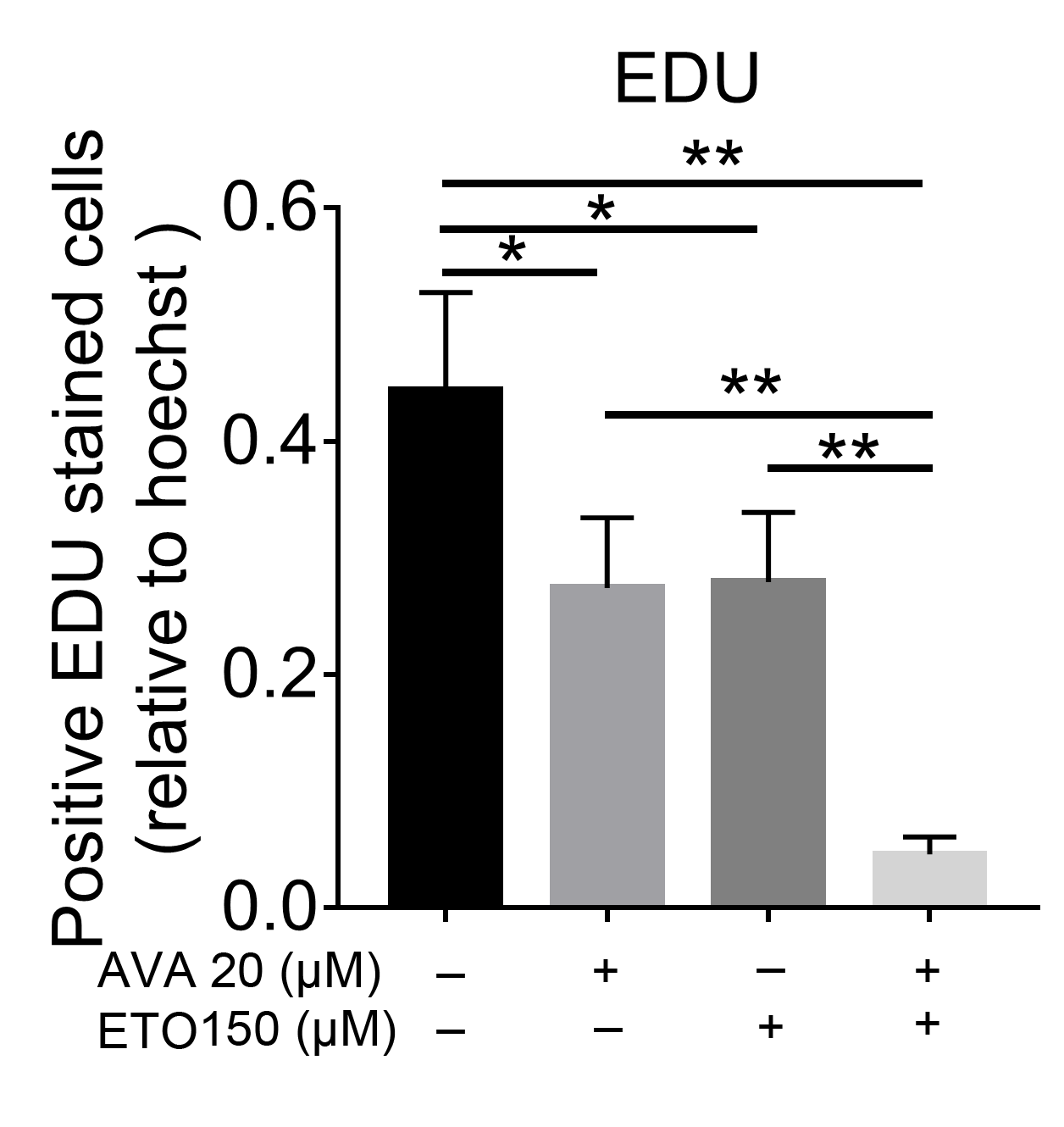

Supplement: Supplementary file 9 — Figure S9 [file 41420_2021_504_MOESM9_ESM.tif]

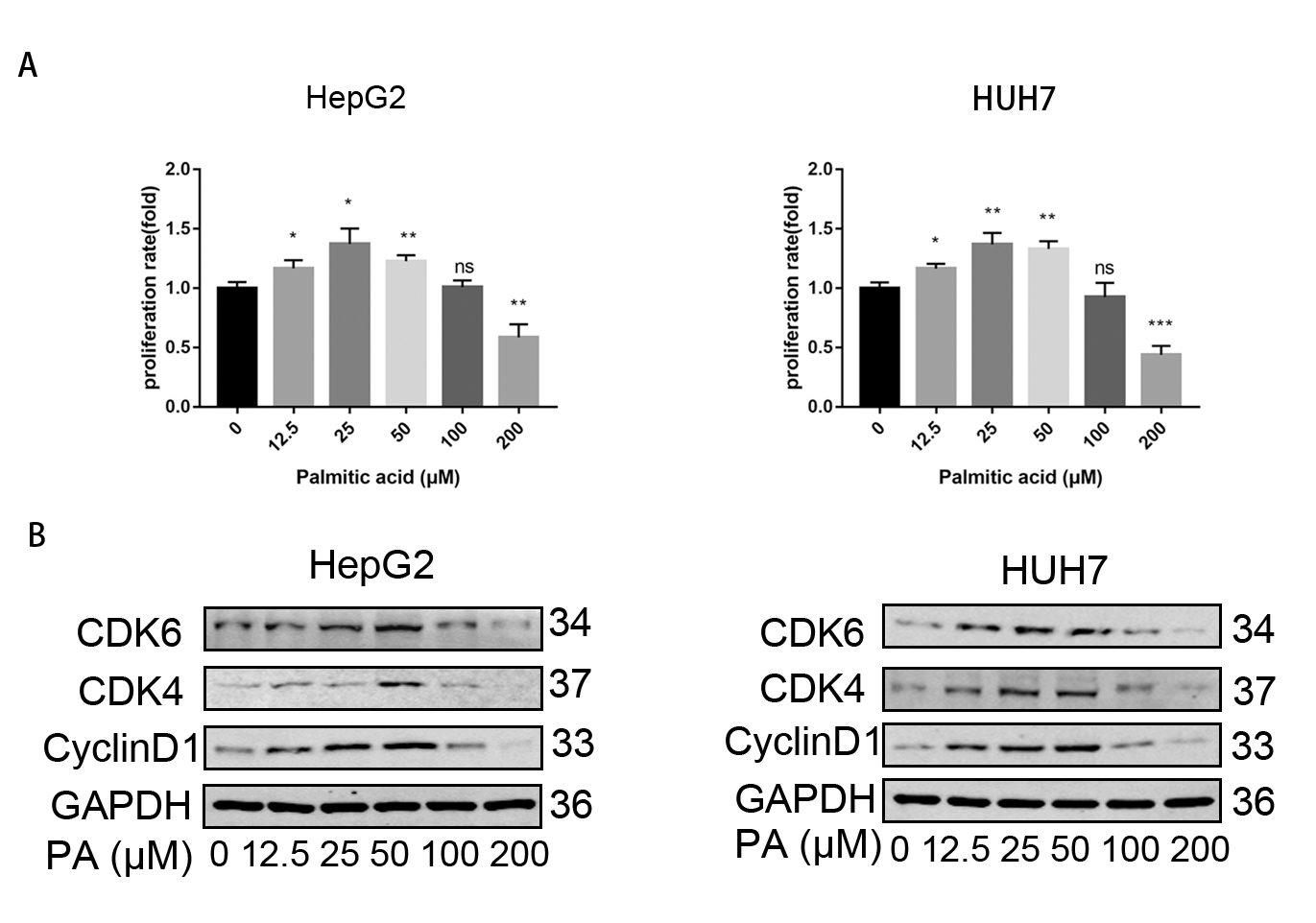

Supplement: Supplementary file 10 — Figure S10 [file 41420_2021_504_MOESM10_ESM.tif]
