# Supplementary material for: Simultaneously targeting SOAT1 and CPT1A ameliorates hepatocellular carcinoma by disrupting lipid homeostasis
Source: Cell Death Discov. 2021 May 29;7:125. doi: 10.1038/s41420-021-00504-1 (PMC8164629; doi:10.1038/s41420-021-00504-1)
Supplement: Supplementary file 11 — Supplementary figure legends [file 41420_2021_504_MOESM11_ESM.doc]

**Figure S1 The overexpression of SOAT1 and CPT1A was independent of DEN administration.**

Representative IHC staining (n = 5 images in total) of SOAT1 or CPT1A in the tissues obtained from ND-fed mice. NT and T indicate non-tumor and tumor areas, respectively (scale bar, 50 μm). Five separate areas from each tissue were quantified (mean ± SEM); *p < 0.05; **p < 0.01, ***p < 0.001.

**Figure S2 palmitic acid (<50 μM）can enhance the expression of SOAT1.**

Western blot analysis of SOAT1 in HepG2 and HUH7 cells treated with palmitic acid at different concentrations.

**Figure S3 Palmitic acid enhances the expression of CPT1A.**

(A-B) Western blot analysis of CPT1A and HMGCL in HepG2 and HUH7 treated with (palmitic acid) PA or OA (oleic acid) at the indicated concentration for 48 h. (C-E) Levels of total acetyl-CoA, ATP, and β-hydroxybutyrate in HCC cells treated with PA or OA for 48 h at indicated concentration (mean ± SED, n = 3); *p < 0.05; ***p < 0.001, ****p < 0.0001; ns: no significance.

**Figure S4 Protein-protein interaction network of SOAT1, FAO, and TCA cycle in STRING database.**

Red nodes: electron transfer activity; green nodes: fatty acid metabolism; blu nodes: Citrate cycle TCA cycle.

**Figure S5 Targeting SOAT1 enhanced the level of free cholesterol.**

(A-B) HUH7 cells were treated with pharmacological (avasimibe, 48 h) or genetic (si-RNA, 72 h) inhibition of SOAT1. Free cholesterol was visualized by Filipin III staining. The white squares are amplified in the right images. Scale bar represents 50 μm, more than 30 cells were analyzed. *p < 0.05.

**Figure S6** A quantification of the grayscale value of HMGCL blots was analyzed by ImageJ software, (means ± SEM, n = 3), *p < 0.05, ***p < 0.001.

**Figure S7 Inhibiting SOAT1 could promote FAO through FFAs.**

(A) HUH7 cells stained with BODIPY after serum starving at different days. (B-C) Fluorescence imaging of LDs stained with BODIPY 493/503 (green) in HUH7 cells treated with pharmacological (avasimibe, 48 h) or genetic (si-RNA, 72 h) inhibition of SOAT1 in serum*-*starved cells*.* Nuclei were stained with DAPI (blue). Scale bar, 50 μm. Data were quantified using ImageJ software; ***p < 0.001, ****p < 0.0001, ns: no significance. (D) Western blot analysis of CPT1A and HMGCL in HepG2 and HUH7 incubated with or without AVA after serum starving.

**Figure S8:** **Kaplan*-*Meier survival curves revealed no significant differences (p = 0.05), but the HFD+AVA group showed a trend toward better survival rates.**

**Figure S9 Quantification of EdU (percentage of EdU+/Hoechst+).**

**Figure S10 Palmitic acids exert different functions in a dose-dependent way**

(A) High doses of PA inhibited cell viability, while the low-dose promoted cell proliferation in HepG2 and HUH7 cells (means ± SEM, n = 3); PA: palmitic acid; *p < 0.05, **p < 0.01, ***p < 0.001. (B) Western blot analysis of CDK4, CDK6, and CyclinD1 in HCC cells treated with PA at the indicated concentration.
